# The *Pseudomonas aeruginosa* Cpx system provides a cyclic-di-GMP independent link between cell envelope stress and surface sensing

**DOI:** 10.1128/mbio.02726-25

**Published:** 2025-12-16

**Authors:** Megan R. O'Malley, Hailey N. Dearing, Xuhui Zheng, Alyssa N. Kretschmer, Timothy H.-S. Cho, Tracy L. Raivio, Matthew R. Parsek

**Affiliations:** 1 Department of Microbiology, University of Washington 312771, Seattle, Washington, USA; 2 Department of Biological Sciences, University of Alberta 98624, Edmonton, Alberta, Canada; Universite de Geneve, Geneva, Switzerland

**Keywords:** *Pseudomonas aeruginosa*, pathogenesis, two-component regulatory systems, biofilms, cell envelope, stress response

## Abstract

Bacteria surveil their cell envelope through a network of envelope stress response systems (ESRs). Beyond regulation of envelope maintenance, ESRs influence expression of a range of virulence traits among pathogenic bacteria. The Cpx two-component system, a conserved ESR, responds to envelope stress generated by bacterial contact with a solid surface. This feature is particularly interesting in the context of bacterial biofilm formation, a key virulence trait of the opportunistic pathogen *Pseudomonas aeruginosa*, which initiates biofilm formation upon detecting envelope stress-related cues of surface adhesion. While a putative Cpx system is present in *P. aeruginosa*, it exhibits dissimilarities from orthologous systems and has not been evaluated for its roles in stress signaling and/or biofilm formation. Here, we found that the *P. aeruginosa* Cpx system includes two previously uncharacterized adaptor protein genes, *cpxM* (*PA3203*) and *cpxH* (*PA3207*), unique to the genus *Pseudomonas*. *P. aeruginosa* Cpx functions as an ESR and is responsive to stimuli related to outer membrane protein dysbiosis. Cpx is also activated upon surface attachment by a mechanism independent of the nucleotide second messenger cyclic-di-GMP (c-di-GMP), a global regulator of *P. aeruginosa* biofilm formation. We further show that the Cpx system influences gene expression related to antibiotic resistance, biofilm matrix production, iron acquisition, and redox homeostasis. These findings present an expanded view of envelope stress signaling in *P. aeruginosa* surface sensing, demonstrating that this biofilm-inducing stimulus is transmitted through both c-di-GMP-dependent and -independent signaling arms.

**IMPORTANCE:**

*Pseudomonas aeruginosa* is an opportunistic bacterial pathogen that causes chronic infection in humans by forming protective, multicellular structures called biofilms. The strong natural resistance of bacterial biofilms to antibiotic and immune clearance presents a major therapeutic obstacle in *P. aeruginosa* disease management. As these structures often assemble on surfaces, i.e. host tissues or indwelling medical devices, the ability of *P. aeruginosa* to sense and respond to surface contact is a key step in initiating biofilm formation. We report that the Cpx signaling system in *P. aeruginosa* is activated upon surface attachment and operates independently of other known surface-sensing systems. Cpx responds to cellular stress, particularly disruptions to cell-surface proteins, suggesting that stress generated by bacterial surface adhesion is a relevant biofilm-inducing signal. These findings expand knowledge of surface-sensing mechanisms in *P. aeruginosa* and link surface recognition to a variety of other disease-related cellular processes regulated by the Cpx system.

## INTRODUCTION

Chronic *Pseudomonas aeruginosa* infection is often associated with the prolific ability of this pathogen to form biofilms on host surfaces, in which sessile, semi-dormant bacteria are embedded in a self-produced extracellular matrix (1, 2). Sensing initial surface adhesion is generally thought to act as an inducing stimulus for bacteria to adopt the biofilm lifestyle (3–6). *P. aeruginosa* senses perturbations to the cell envelope generated by surface contact, which stimulates a rapid increase in the intracellular concentration of the signaling molecule cyclic-di-GMP (c-di-GMP) (7, 8). C-di-GMP is a global biofilm regulator that suppresses cellular motility and stimulates matrix production. The molecular stress-related signal(s) arising from surface adhesion are not fully understood, though stimuli including contact-induced membrane deformation, disruption of cell envelope protein folding, and/or physiochemical properties of the surface environment which affect envelope function (i.e., local pH or osmolarity) have been discussed as potential mechanisms (9, 10). A broader exploration of *P. aeruginosa* surface-sensing mechanisms is currently limited by the fact that envelope stress signaling in this pathogen has not been extensively characterized, with most molecular knowledge derived from enteric model organisms such as *Escherichia coli*.

The Gram-negative cell envelope, consisting of an inner and outer membrane with an intervening peptidoglycan cell wall, is intrinsic to bacterial cell integrity and survival. Envelope homeostasis is impacted by various stressors inherent to the host environment, such as changes in temperature and redox potential encountered during infection, and is also affected by several classes of antibiotics (11). In the model organism *E. coli*, a network of envelope stress response systems (ESRs) monitors envelope integrity (e.g., the σ^E^, Cpx, Bae, Rcs, and Psp systems) (12). These ESRs play somewhat specialized roles in sensing distinct stimuli arising from envelope stress; for example, misfolded outer membrane proteins are detected by σ^E^, while cell-surface lipopolysaccharide (LPS) defects trigger the Rcs system (13, 14). In addition to remediating envelope damage repair, ESRs have been found to elicit broader effects on the cell, including regulation of virulence gene expression (15).

The conserved Cpx ESR is particularly notable for its effects on virulence. Discovered in *E. coli*, CpxRA is a classic two-component system comprised of an inner membrane sensor histidine kinase CpxA, which modulates the activity of an OmpR-type response regulator CpxR through phosphorylation (16). The CpxA sensor integrates multiple stress-related signals in *E. coli*, including interactions with envelope proteins that are misfolded or aberrantly localized at the inner membrane due to defects in protein homeostasis and/or trafficking (17–22). Accordingly, the first identified regulatory targets of CpxR in *E. coli* were “quality control” factors (i.e., *spy*, *dsbA*, *ppiA*, *degP*/*htrA*) involved in remediating or recycling envelope proteins (23–26). Cpx-mediated tolerance of envelope stress is essential for various pathogenic and mutualistic bacterial species to survive within host systems (27–30). The Cpx response has since been found to modulate the expression of a wide variety of genes in many Proteobacteria, including virulence factors such as flagella, pili, chemotaxis systems, biofilm matrix components, exotoxins, and secretion systems (31–35). Both direct transcriptional regulation by CpxR and indirect effects on the stability of large envelope-localized protein complexes due to Cpx-regulated homeostasis factors influence virulence factor biogenesis (36–38). As such, Cpx-associated virulence phenotypes are often complex and vary widely across systems.

*E. coli* Cpx is also induced by cellular surface adhesion, making this system a compelling target for study in the context of *P. aeruginosa* surface sensing and biofilm formation. While a putative homologous Cpx ESR has been identified in *P. aeruginosa* (39), its roles in stress signaling and virulence have not been fully determined. However, spontaneous adaptive mutations in the Cpx sensor kinase are frequently recovered from clinical and antibiotic resistance-evolved populations of *P. aeruginosa*, suggesting pathogenic relevance of this system (40–44). Several of these mutants, predicted to confer constitutive activation of Cpx signaling, exhibit enhanced antibiotic resistance due to elevated expression of cellular efflux pumps, which are directly regulated by Cpx in both *P. aeruginosa* and *E. coli* (39, 45–47). Transcriptomic studies have also reported elevated Cpx-associated gene expression in *P. aeruginosa* upon surface attachment, including attachment to a variety of abiotic surfaces (48) and pulmonary epithelial tissue (49).

In the present study, we characterize the *P. aeruginosa* Cpx system. *P. aeruginosa* encodes two previously uncharacterized, *Pseudomonas*-specific proteins within the conserved *cpx* locus, including a putative inner-membrane adaptor protein CpxM (PA3203) required for full Cpx system activity. We demonstrate that Cpx signaling is activated under cell envelope stress, likely related to an outer membrane protein-related signal. Through analysis of genomic CpxR promoter-binding sites and transcriptomic profiling, we find that the PAO1 Cpx response impacts cellular efflux, biofilm matrix production, and iron acquisition and participates in complex regulation of energy metabolism. The *P. aeruginosa* Cpx regulon notably lacks many of the canonical Cpx targets involved in envelope protein homeostasis previously identified in *E. coli*, suggesting diversification of Cpx stress signaling in this organism. Our findings further demonstrate that the Cpx surface response constitutes a c-di-GMP-independent surface-sensing mechanism.

## RESULTS

### Genetic analysis of the Cpx two-component system reveals multiple putative adaptors that impact signaling

**Fig 1:**
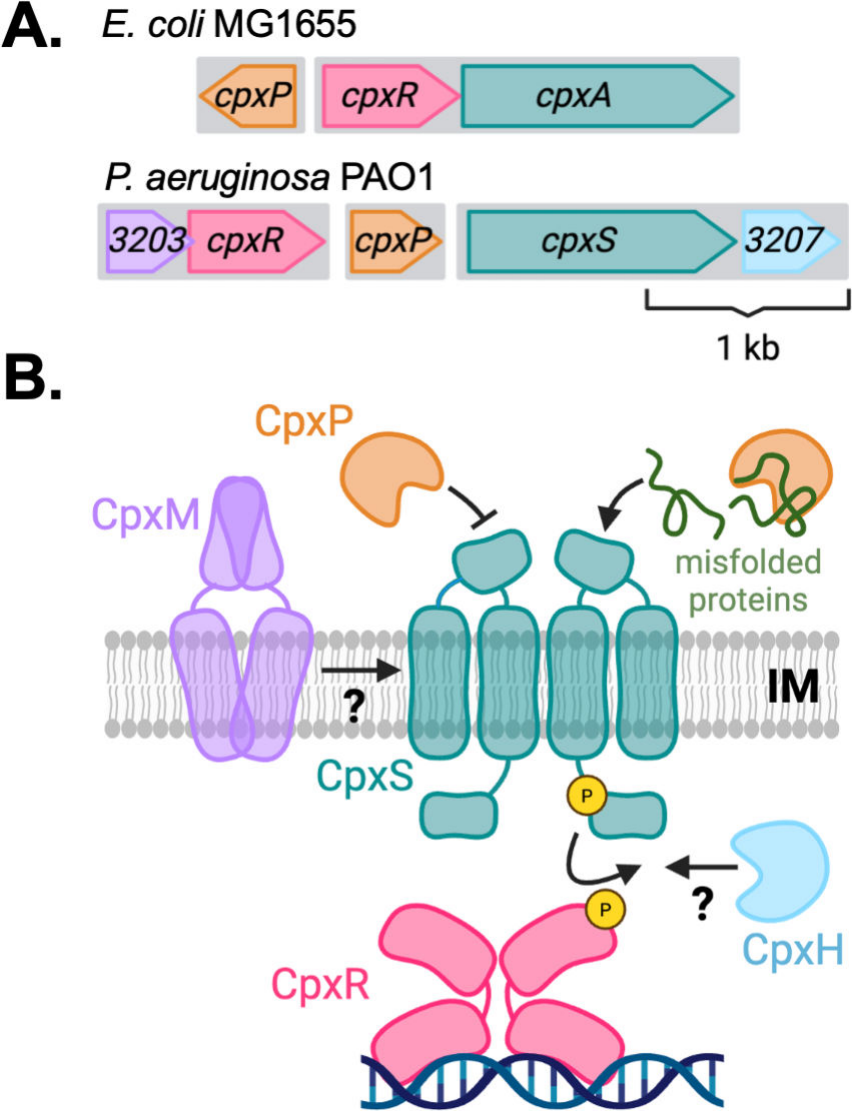
An extended Cpx system in *P. aeruginosa*. (**A**) Schematic of the *cpx* genetic locus from *E. coli* MG1655 and *P. aeruginosa* PAO1 to approximate scale. Cpx protein-coding sequences are indicated by colored arrows in the direction of transcription. Shaded boxes indicate transcriptional units as inferred for the *E. coli* Cpx system (50) and predicted by Rockhopper for PAO1 (53). Hypothetical protein-coding genes c*pxM* and *cpxH* were previously annotated as *PA3203* and *PA3207* in PAO1, respectively. Scale bar indicates 1 kb. (**B**) Hypothetical model of Cpx complex formation and envelope stress signaling in PAO1. Two-component sensor histidine kinase CpxS (teal) phosphorylates response regulator CpxR (magenta) from the inner membrane (IM), stimulating CpxR binding to regulatory DNA targets. Repressor protein CpxP modulates signaling by interacting with the periplasmic signaling domain of CpxS, and/or through chaperone activity on misfolded proteins in the periplasm. Prospective interactions between CpxM/H and the two-component system are indicated. Graphics were generated using BioRender.

The Cpx system consists canonically of a sensor histidine kinase, annotated either CpxA or CpxS by species convention, a response regulator CpxR, and a conserved periplasmic adaptor protein, CpxP. Homologous Cpx systems are present throughout the α-, β-, and γ-Proteobacteria classes. Among representative genomes, the majority of *cpx* loci exhibit an organization similar to *E. coli* K-12, in which the *cpxP* gene is encoded in diverging orientation from an inferred *cpxAR* bicistronic operon (Fig. 1A) (50). By contrast, in the Pseudomonadaceae family, *cpxS* and *cpxR* are encoded separately on opposite sides of *cpxP* (Fig. S1). Cpx homologs in the model isolate *P. aeruginosa* PAO1 are encoded by *PA3204* (CpxR, 64.5% protein similarity to *E. coli* K12 MG1655), *PA3205* (CpxP, 35.6%), and *PA3206* (CpxS, 43.4%). Within the *Pseudomonas* genus exclusively, additional genes are associated with the *cpx* locus (Fig. 1A; Fig. S1). Hypothetical protein-coding gene *PA3203* forms an operon with *cpxR* and is generally conserved throughout the genus *Pseudomonas*, with few exceptions (i.e., *Pseudomonas putida* KT2440). An additional protein-coding gene, *PA3207*, is present exclusively within the *P. aeruginosa* species cluster in an operon with *cpxS. PA3203-cpxR* and *cpxS-PA3207* operons were predicted by the Database of Prokaryotic Operons (DOOR, Pseudomonas Genome Database) (51, 52), and validated using Rockhopper operon analysis (53) from PAO1 RNA-Seq data generated in this study (Table S1).

PA3203 is a small inner membrane protein, predicted by AlphaFold2 to harbor two linked transmembrane and periplasmic α-helices which homodimerize (54). PA3207 has a predicted α/β fold structure similar to those found among bacterial histidine kinase catalytic/ATP-binding domains (55), and harbors a potential catalytic histidine residue (His72). Models of PA3207 exhibit a disordered N-terminal domain consistent in size (21aa residues) to a typical signal peptide; however, this sequence does not match any known signal peptide consensus sequences (SignalP 6.0, PSORTb), and full-length PA3207 was predicted to localize to the cytoplasm by the MatureP classifier (56–58). Based on these predictions, we hereafter refer to PA3203 as CpxM (Cpx membrane protein) and PA3207 as CpxH (Cpx histidine kinase-like protein).

**Fig 2:**
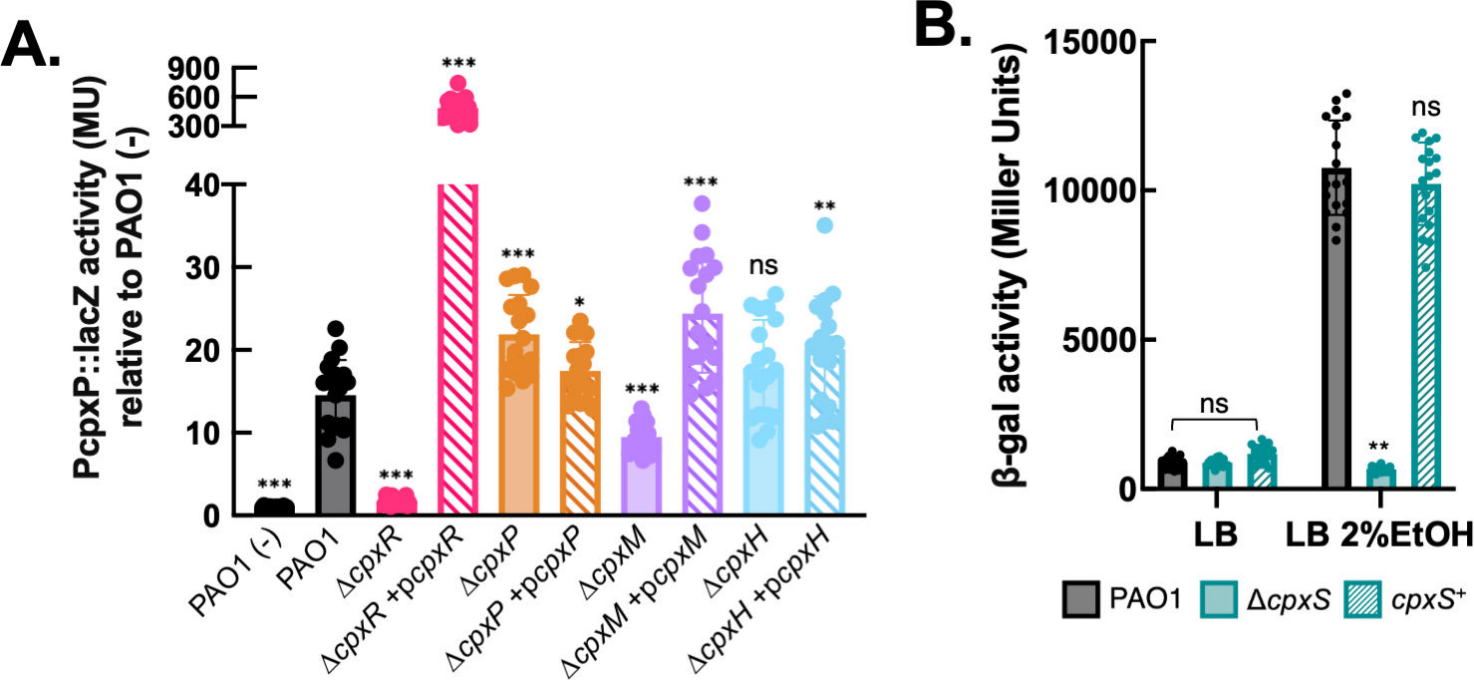
The PAO1 *cpx* locus encodes a multi-adapter two-component regulatory system. (**A**) Genetic analysis of the PAO1 Cpx signaling system. Activity of a P*_cpxP_::lacZ* transcriptional reporter integrated at the neutral Tn*7* site was measured in PAO1 and *cpx* gene deletion mutants, expressing either an unmodified pJN105 plasmid (Gm^R^) or pJN105 expressing complementing alleles of *cpx* genes under arabinose-inducible control. Reporter activity was quantified by standard β-galactosidase assay (Miller units, MU) on cell lysates from bacterial cultures grown to exponential phase (4 h) in the presence of 0.1% arabinose and 100 µg/mL gentamicin. Cellular stress was induced by the addition of 2% ethanol. Graphed are means (± standard deviation, SD) of P*_cpxP_* reporter activity relative to a baseline level defined by PAO1 grown in unstressed LB (-). Asterisks denote statistical significance based on pairwise *t*-tests between mutant and ethanol-stressed PAO1 reporter activity levels, ***P < 0.001, **P < 0.01, *P < 0.05, “ns” no significant difference. Data are pooled from three independent experiments; *n* = 18. (**B**) Complementation of P_cpxP_::*lacZ* reporter activity in a PAO1 ∆*cpxS* mutant by restoring the wild-type allele to the chromosome through allelic exchange (*cpxS*^+^). Mean (± standard deviation, SD) MU are shown. Asterisks denote statistical significance based on pairwise *t*-test between mutant and wild-type PAO1 reporter activity levels, ***P > 0.001, “ns” no significant difference. Data are pooled from three independent experiments; *n* = 18. Graphing and statistical analysis were performed in GraphPad Prism v10.1.

*P. aeruginosa* CpxR has been previously shown to interact with a “Cpx box” promoter motif originally identified in *E. coli* and regulates the transcription of target genes including *cpxP*. As *cpxP* promoter activity has been widely used as a readout of Cpx signaling in other systems (59–62), we evaluated the activity of a chromosomal P*_cpxP_::lacZ* transcriptional reporter in PAO1 and *cpx* gene deletion mutants. While basal activity of this reporter was low in unstressed LB growth medium (Fig. S2), activity was stimulated ~15-fold during growth in the presence of 2% ethanol, a known Cpx inducer in *E. coli* (61). This response was fully abrogated in both PAO1 ∆*cpxR* and ∆*cpxS* mutants (Fig. 2). Complementation of the ∆*cpxR* mutant by plasmid-based overexpression of *cpxR* drastically increased reporter activity, likely due to the high dosage of the complementing allele as noted in previous studies (63, 64). By contrast, the PAO1 ∆*cpxS* stress response could not be restored by plasmid-based overexpression *cpxS* allele (data not shown). We hypothesize that overexpression of CpxS may lead to an accumulation of the sensor kinase in its inactive conformation, as CpxS/CpxA orthologs exhibit dual kinase/phosphatase activity toward CpxR (65); as sensor kinases depend on homodimerization for catalytic activity, perturbations to the stoichiometry of the system by overexpression may further complicate plasmid-based complementation. As an alternative approach, we used allelic exchange to reintroduce the wild-type *cpxS* allele to the PAO1 ∆*cpxS* mutant strain. The resulting strain, PAO1 *cpxS^+^*, exhibited a fully restored Cpx response (Fig. 2B). We note that PAO1 ∆*cpxS* displays slightly elevated basal activity in unstressed media when assessed with an alternative reporter system (pBBR1 P*_cpxP_*::mGreenLantern/P*_rpoD_*::mScarlet-I, described in later sections), which is more sensitive to heterogeneous patterns of gene expression within a population, consistent with the notion that CpxS may exert complex positive and negative regulation of Cpx signaling in *P. aeruginosa* as in other bacterial systems.

Using this reporter system, we similarly assessed the role(s) of CpxP and the hypothetical proteins CpxM and CpxH. In *E. coli*, CpxP binds misfolded periplasmic proteins and the sensor domain of CpxA, functioning largely as a negative autoregulator of Cpx activity (59, 66, 67). CpxP is the only PAO1 homolog to *E. coli* CpxP and its paralog, Spy (66, 68); we note that *P. aeruginosa* CpxP exhibits stronger protein similarity to Spy (47.4%) than to *E. coli* CpxP. A PAO1 ∆*cpxP* mutant displays elevated reporter activity, indicating that, as expected, CpxP dampens Cpx activity in *P. aeruginosa*. Complementation of PAO1 ∆*cpxP* restored near wild-type levels of activity. PAO1 ∆*cpxP* did not exhibit constitutive activation during unstressed growth in LB (Fig. S2), suggesting that de-repression in the absence of CpxP is not alone sufficient to activate the Cpx system. Meanwhile, a PAO1 ∆*cpxM* mutant strain displayed a significantly reduced response under ethanol stress, which was restored by *cpxM* complementation. A PAO1 ∆*cpxH* mutant strain exhibited an ethanol response similar to wild-type PAO1, though *cpxH* plasmid-based complementation significantly elevated reporter activity, suggesting that CpxH is nonessential but may play an accessory role in enhancing Cpx signaling. We note that plasmid-based expression of complementing *cpx* gene alleles significantly elevated Cpx reporter activity above PAO1 levels, which was likely an effect of gene dosage due to plasmid copy number and/or gene expression levels under inducible promoter control. Our genetic characterization of the PAO1 Cpx system suggests that the core CpxRS two-component system is required for activation of the Cpx stress response, while accessory proteins CpxM and CpxP function, respectively, as positive and negative regulators of Cpx activity.

### The PAO1 Cpx system functions as a cell envelope stress sensor

Several envelope stress-related stimuli, including alterations in membrane composition and toxic protein accumulation, have been previously shown to induce Cpx signaling in various bacteria (18, 69–71). To determine the Cpx stimulon in *P. aeruginosa*, we screened a variety of chemical stressors with known impacts on envelope homeostasis. To effectively compare the impact of various stressors on Cpx signaling, we utilized chemical concentrations that uniformly suppressed growth rate to around 20% of unstressed PAO1 growth (Fig. S3). Cpx activity in PAO1 was evaluated by a pBBR1 plasmid-based bicolor fluorescence reporter containing the transcriptional fusions P*_cpxP_*::mGreenLantern (mGL) and P*_rpoD_*::mScarlet-I, the latter a constitutively active reporter used to normalize P*_cpxP_* reporter signal (6, 8). Fluorescence intensity was quantified from single cells imaged by microscopy. We found P*_rpoD_*::mScarlet-I reporter activity to be consistent across stress conditions, including those that impacted cell growth (Fig. S4 and S9).

**Fig 3:**
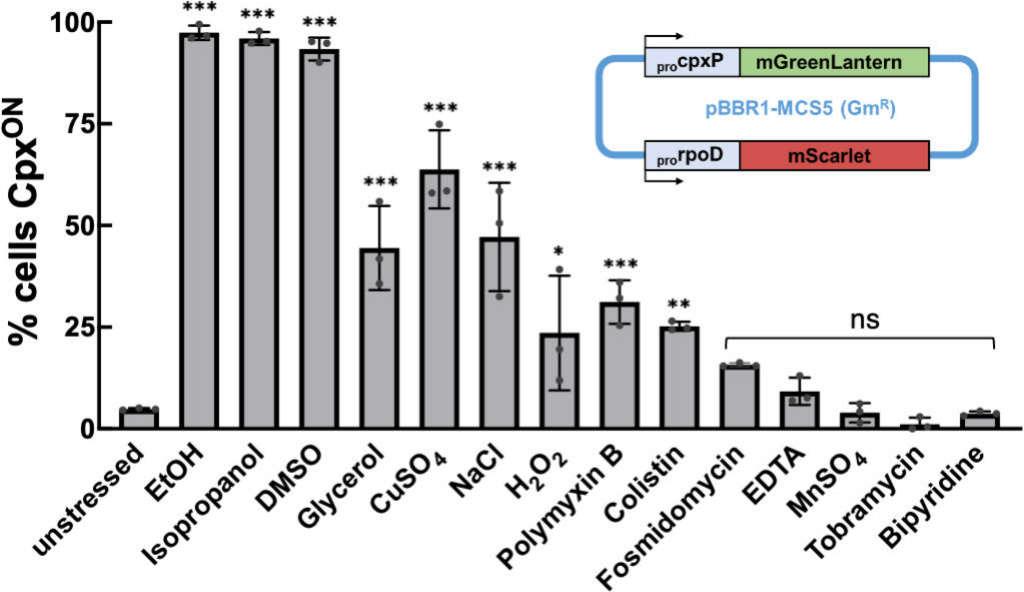
Chemicals that disrupt envelope homeostasis impact Cpx signaling. Activity of a bicolor fluorescent reporter plasmid (inset), containing transcriptional fusions of P*_cpxP_*::mGreenLantern (mGL) and the housekeeping promoter P*_rpoD_*::mScarlet, was assessed in PAO1 after 4 h of growth in the presence of select chemical stressors. Fluorescence intensity was quantified from single bacterial cells imaged by microscopy. Normalized mGL/mScarlet values were compared to those of unstressed cells; cells were considered Cpx^ON^ when normalized reporter activity exceeded twofold the mean value from the unstressed cell population. Graphed are means (± SD) of the percentage of Cpx^ON^ cells in the imaged population from data pooled across three independent experiments, with >100 total cells imaged per condition. Chemical stressors were applied at the following concentrations: 2% (vol/vol) ethanol, 1% (vol/vol) isopropanol, 4% (vol/vol) DMSO, 8% (wt/vol) glycerol, 3 mM CuSO_4_, 400 mM NaCl, 12.5 mM hydrogen peroxide, 0.5 µg/mL polymyxin B, 0.38 µg/mL polymyxin E (colistin), 32 µg/mL fosmidomycin, 0.3 mM EDTA, 5 mM MnSO_4_, 4 µg/mL tobramycin, and 1.4 mM bipyridine. One-way ANOVA with multiple comparison test was performed in GraphPad Prism v10.3.0; *P < 0.05, **P < 0.01, ***P < 0.001, “ns” no significant difference.

Through this approach, we identified a variety of stressors that elicit the Cpx response in PAO1 (Fig. 3). As P*_cpxP_*::mGL reporter activity showed significant heterogeneity within the population, we scored the impact of stressors based on the percentage of individual cells with high Cpx reporter activity (Cpx^ON^) based on an experimentally determined threshold (see Materials and Methods) (72). Protein denaturants such as ethanol, isopropanol, and DMSO were the strongest inducers, with over 90% of cells designated Cpx^ON^. Other stressors exerted milder but significant impacts on Cpx activity, including oxidative and osmotic stress. Reporter activity was also stimulated by polymyxins, a class of antibiotics that impact outer membrane integrity by disrupting cell-surface LPS stability (73), suggesting that outer membrane dysbiosis is also relevant to Cpx stress signaling. Importantly, we observed that multiple stressors that impacted PAO1 cell growth did not elicit a significant Cpx response, demonstrating that this response was not simply attributable to a general stress response or altered growth.

**Fig 4:**
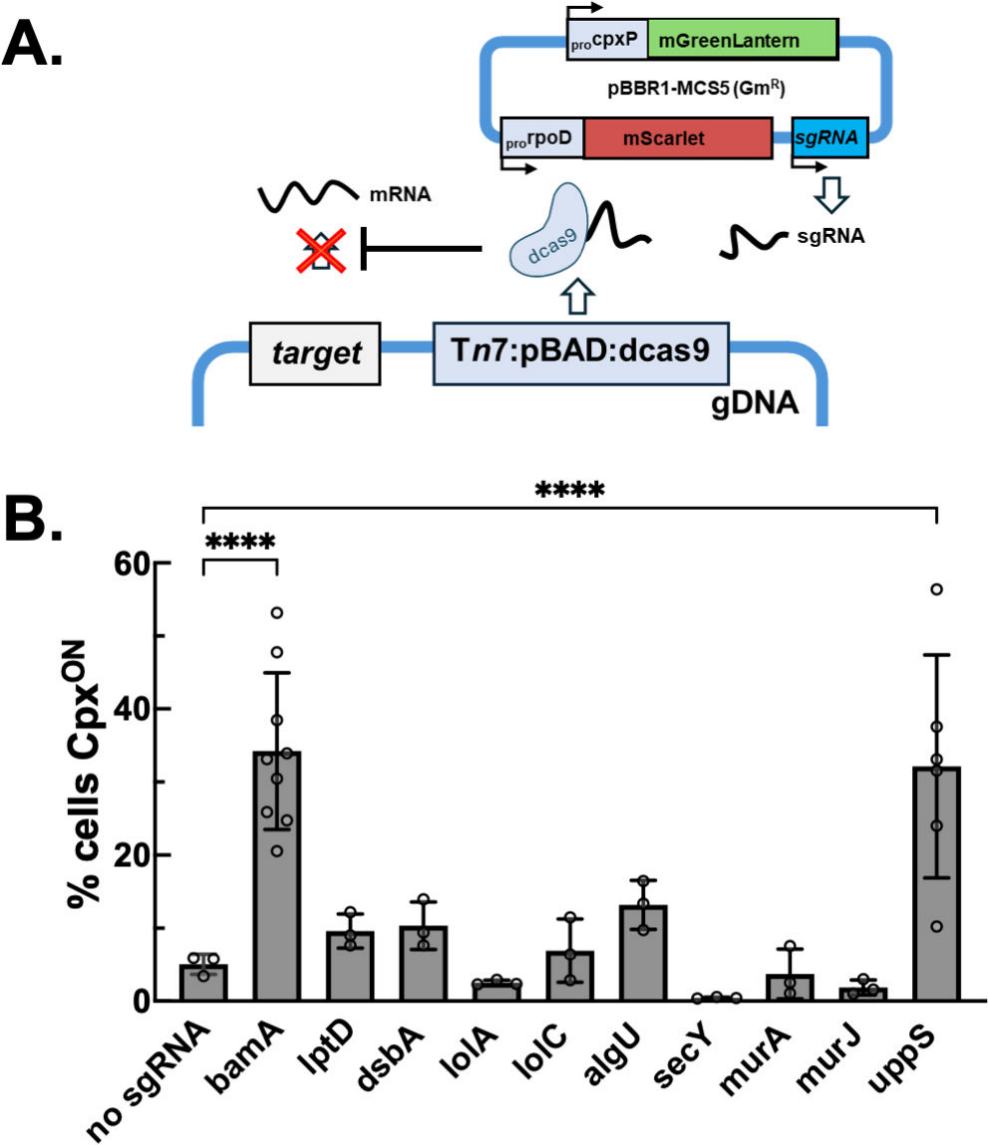
CRISPRi knockdown of key envelope homeostasis regulatory genes impacts Cpx signaling. (**A**) Schematic of the CRISPRi experimental approach. PAO1 strains with an integrated, Tn*7::araBAD::sp*dcas9 cassette expressed a bicolor fluorescent reporter pBBR1 plasmid, containing transcriptional fusions of P*_cpxP_*::mGreenLantern and P*_rpoD_*::mScarlet-I. sgRNAs targeting the indicated genes were constitutively expressed under SpeI promoter control from the same plasmid. (**B**) Cells (>100 per condition) were imaged by microscopy after 4 h incubation with 0.2% arabinose to induce *sp*dcas9 expression. Single-cell fluorescence intensity quantified with MicrobeJ and normalized P*_cpxP_*::mGreenLantern/ P*_rpoD_*::mScarlet values were compared to those from cells expressing a fluorescent reporter plasmid with no sgRNA. Cells were considered Cpx^ON^ when normalized reporter activity exceeded twofold the mean value from the control population (no sgRNA). One-way ANOVA with multiple comparisons test was performed in GraphPad Prism v10.3.0; ****P < 0.001. Data are pooled from three biological replicates, except for the *bamA* and *uppS* conditions, which represent 9 and 6 biological replicates, respectively.

While these findings substantiated that the PAO1 Cpx system generally responds to cell envelope stress, the pleiotropic effects of many chemical stressors on the cell envelope limit the interpretation of these results. We therefore employed an additional CRISPRi approach to conditionally deplete expression of genes involved in cell envelope homeostasis. This system is comprised of an integrated *sp*dcas9 enzyme under inducible promoter control; when recruited to target genes by a single guide RNA (sgRNA) probe, *sp*dcas9 inhibits transcript elongation by steric hindrance (Fig. 4A) (74, 75). To assess the impact of CRISPRi gene knockdown on Cpx signaling, sgRNAs were expressed from the same pBBR1 plasmid encoding P_cpxP_::mGL / P_rpoD_::mScarlet-I reporters. Induction of *sp*dcas9 expression in the absence of a functional sgRNA did not impact growth, indicating that this system is not toxic to producer cells (Fig. S5 and S6). We screened a panel of sgRNAs, spanning various essential cell envelope functions including general secretion (*secY*), periplasmic protein folding (*dsbA*), envelope stress signaling (*algU*), LPS biogenesis (*lptD*, *uppS*), outer membrane protein folding (*bamA*), lipoprotein trafficking (*lolA, lolC*), and peptidoglycan biosynthesis (*murA*, *murJ*, *uppS*). The effectiveness of CRISPRi gene depletion was validated by the severe growth phenotypes incurred when *sp*dcas9 was induced in the presence of sgRNAs targeting essential functions and/or phenotypic effects of sgRNA targeting for non-essential genes (*dsbA* and *algU*), (Fig. S7).

Most sgRNAs did not induce Cpx signaling (Fig. 4B; Fig. S8). However, we observed a significant increase in Cpx activity when either the *bamA* or the *uppS* gene expression was depleted by CRISPRi. BamA is a subunit of the conserved Bam complex, which mediates the insertion of β-barrel proteins into the outer membrane (76). UppS is a cytoplasmic enzyme that catalyzes the formation of undecaprenyl pyrophosphate (often referred to as C55-PP). C55-PP is a precursor to lipid II, an inner membrane “carrier lipid” which mediates the export of hydrophilic envelope components, including peptidoglycan and LPS (77, 78). However, we did not observe Cpx induction by CRISPRi attenuation of other genes involved in cell wall or LPS biogenesis (*lptD*, *murA*, *murJ*), suggesting that defects in the cell wall and/or cell surface LPS are not alone sufficient for Cpx activation. While these findings substantiate a role of Cpx in envelope stress signaling, further study is necessary to determine the exact molecular signal(s) produced by *bamA* and *uppS* depletion responsible for Cpx activation.

### The Cpx system regulates gene expression related to efflux, iron acquisition, adhesion, and redox stress in PAO1

Around 100 promoters have been identified as targets of CpxR in *E. coli* based on the presence of a CpxR conserved direct-repeat DNA-binding motif (5′-GTAAA-N_5_-GTAAA-3′), which the *P. aeruginosa* PA14 CpxR homolog binds to *in vitro* (26, 39, 79). We queried the PAO1 genome for CpxR motifs present in gene promoter regions and identified 37 transcriptional units in PAO1 potentially under Cpx regulation (Table 1). Prospective CpxR-regulated genes in PAO1 included previously established CpxR targets in *E. coli*, including *cpxP*, the efflux system *muxABC-opmB*, a putative cytochrome b561 *yceJ* (*PA3575*), and NADH dehydrogenase *nuoAB* (61, 80, 81). However, PAO1 orthologs of many canonical *E. coli* CpxR targets (*dsbA*, *ppiA*, *algU* [*rpoE*], *algW* [*degP*/*htrA*], *PA2604* [*yccA*], *PA2830* [*htpX*], and *PA3712* [*yebE*]) lacked any evident CpxR promoter-binding sites, suggesting that the Cpx regulon may differ substantially between these organisms.

**TABLE 1:** Predicted CpxR-binding sites in PAO1^*c*^

Locus	Gene	Gene function	Dist. from ATG	Predicted CpxR site	ID motif	ID DAP-Seq
PA0425	mexA	RND efflux protein	−246	GTAAACCTAATGTAAA	y	y
PA0502	nirQ	Transcriptional regulator, denitrification	−87	GTCAAGCAAGGGTAAA	y	n
PA0527	dnr	Transcriptional regulator, denitrification	−129	GTAAGCCTTGGCTTAC^*a*^	n	y
PA0546	metK	Methionine adenosyltransferase	−86	GTAAACCAATGTAAA	y	n
PA0865	hpd	4-Hydroxyphenylpyruvate dioxygenase	−194	GTAAAGATAACTTTAC^*a*^	n	y
PA0873	phhR	Transcriptional regulator, phenylalanine catabolism	−124	GAATTGGCCTGGGTCGC ^*a*^	n	y
PA0888	aotJ	Periplasmic arginine transport protein	−258	GTAATCAATGAGTAAA ^*a*^	n	y
PA0931	pirA	Ferric enterobactin outer membrane receptor	−195	GGCCTGGATGTAAATG ^*a*^	n	y
PA1003	mvfR	Transcriptional regulator, PQS quorum sensing	−366	GTTAAATAACCGGTAAA ^*a*^	n	y
PA1156	nrdA	Ribonucleotide reductase	−196	GTAAACAGACAGTCAA	y	n^*b*^
PA1097	fleQ	Transcriptional regulator, flagellar motility/adhesion	−212	GTTTTGGGGGATATGTAAA ^*a*^	n	y
PA1345		Hypothetical ATP-binding protein	−69	GTAAAGCTTGCGTAA	y	n
PA1528	zipA	Inner membrane cell division protein	−147	GTGTCAGGCAGTGTAAA ^*a*^	n	y
PA2017		Hypothetical inner membrane beta-propeller protein	−164	GTAAAGGTAAA ^*a*^	n	y
PA2231	pslA	Exopolysaccharide psl biosynthesis protein	−181	GTAGTCTGGGTAA ^*a*^	n	y
PA2523	czcR	Two-component response regulator, metal sensing	−22	GATTCATTTGCAAGTAAA ^*a*^	n	y
PA2528	muxA	RND efflux protein	−78,−89	GTAAATAGCGGGTAAA, GTAAAGCAAGGGTAAA	y	y
PA2558		Hypothetical inner membrane MgtC-like transport protein	−105	GTAAACATGCTCTTC ^*a*^	n	y
PA2637	nuoA	NADH dehydrogenase I subunit	−363	GTAAACAATAGTAA	y	n
PA2936		Cytochrome b561	−325	CAAGCTGACACAGATGTAA ^*a*^	n	y
PA3064	pelA	Exopolysaccharide pel biosynthesis protein	−147	GCTAATTGCTAAA ^*a*^	n	y
PA3155	wbpE	Lipopolysaccharide biosynthesis protein	−317	GTAAACGTTGGTAAA	y	n
PA3205	cpxP	Periplasmic cpx repressor protein	−81	GTAAAGCTTGGGTAAA	y	y
PA3268		Siderophore outer membrane receptor	−53	GTAAAGAACGTAAA	y	y
PA3575	yceJ	Cytochrome b561	−63,−74	GTCAAGAACGGGTAAA, GTAAAGGCGGAGTAAA	y	y
PA3762		Hypothetical metal-binding protein	−97	GTAAAGGCAGCGTCAA	y	y
PA3795		Cytoplasmic oxidoreductase	−144	GTAAAGCTTCAGTAAA	y	y
PA3965		Transcriptional regulator	−315,−326	GTAAACGCCGGGTAAA, GTAAAGAATGGGTAAA	y	y
PA3978		Hypothetical SEL1 repeat family protein	−93	GTAAAGACTTTGTAA	y	y
PA4075		Hypothetical methyltransferase	−61	GCATTCCGGCCAATGTAAA ^*a*^	n	y
PA4168	fpvB	Ferric pyoverdine outer membrane receptor	−111	GTAAAAATGGAGTAAA	y	y
PA4315	mvaT	Transcriptional regulator, exotoxin production	−66	CGAAACTGTAAGTAAA ^*a*^	n	y
PA4431		Hypothetical inner membrane iron-sulfur protein	−274	GTAAAGCGCGTAA	y	n
PA4513	piuC	Inner membrane siderophore oxidoreductase	−131	GTAAATAGAGCGTAAA	y	y
PA4514	piuA	Siderophore outer membrane receptor	−91	GTAAAGAATGTAAA	y	y
PA4628	lysP	Inner membrane lysine permease	−148	GTAAAAAGCTGTAA	y	n
PA4798		Hypothetical protein	−368	GTAAAACCCTACGTCAA	y	n

^*a*^ Indicates binding sites estimated based on DAP-Seq peak location, which typically contained a partial CpxR consensus motif.

^*b*^ Indicates a site which was predicted by DAP-Seq (for the *PA1156* gene promoter) but fell below the 10x enrichment cutoff we imposed on our analysis. Prospective gene functions were assigned based on annotations from the Pseudomonas Genome Database and databases curated by UniProtKB.

^*c*^ CpxR sites within putative gene promoter regions in PAO1 were curated based on a motif search for CpxR consensus and near-consensus sequences using the Pseudomonas Genome Database (ID motif, yes/no), and high-likelihood CpxR-binding sites identified by DAP-Seq in a previous study (DAP-Seq, yes/no) (52, 82).

To investigate the Cpx regulon, we profiled the global transcriptome of a PAO1 strain with a Cpx-activating mutation in the sensor kinase CpxS (PAO1 *cpxS*^T163P^). A spontaneous missense mutation in CpxS was originally identified in an *in vitro* evolved isolate of *P. aeruginosa* PA14 under antibiotic stress (40). Adaptive *cpxS* mutations have been frequently identified in various stress-evolved and host-derived *P. aeruginosa* populations, many of which similarly confer transmembrane proline substitutions (43, 44). We introduced this mutation into our PAO1 wild-type strain and found that it drastically elevated P*_cpxP_::lacZ* reporter activity in a *cpxR*-dependent manner (Fig. S10). Using RNA-Seq to identify differentially expressed genes (DEGs) between PAO1 vs. PAO1 *cpxS*^T163P^, we identified a total of 176 DEGs (*P*_adj_ <0.01), with 110 upregulated and 66 downregulated in PAO1 *cpxS*^T163P^ (Table 2; Table S2) (83). qRT-PCR was used to validate the expression level of select DEGs (Fig. S11). Several predicted direct targets of CpxR were upregulated in PAO1 *cpxS*^T163P^, including *cpxP*, the efflux systems *mexAB-oprM* and *muxABC-opmB*, *czcS*, *yceJ* (*PA3575*), *PA3795*, *piuC* (*PA4513*), and genes from the *pel* exopolysaccharide operon. These results coincide with previous findings that the Cpx response upregulates the *mexAB-oprM* and *muxABC-opmB* efflux systems in both *E. coli* and PA14. Cpx regulation of *pel*, a biofilm matrix component in *Pa*, also echoes other studies in which the Cpx system has been found to regulate a variety of cell-surface adhesins (31, 33, 35, 84, 85).

**TABLE 2:** Select DESeq2 DEGs in a PAO1 *cpxS*^T163P^ activating mutant^*b*^

Locus	Gene	Description	log2FC	*P*_adj_
DEGs upregulated in PAO1 *cpxS*^T163P^				
Cpx locus				
PA3205^*a*^	cpxP	cpx periplasmic accessory protein	8.79	<0.001
PA3206	cpxS	Sensor histidine kinase	5.48	<0.001
PA3207	cpxH	cpx accessory protein	3.88	<0.001
PA3208	ydjA	NADPH nitroreductase	2.65	<0.001
PA3209	ykgJ	Cysteine cluster protein	1.56	<0.001
Efflux				
PA2525^*a*^	opmB	Outer membrane efflux protein	3.37	<0.001
PA2526^*a*^	muxC	RND efflux protein	3.36	<0.001
PA2527^*a*^	muxB	RND efflux protein	3.53	<0.001
PA2528^*a*^	muxA	Efflux membrane fusion protein	3.97	<0.001
PA0425^*a*^	mexA	Efflux membrane fusion protein	1.85	<0.001
PA0426^*a*^	mexB	RND efflux protein	1.64	<0.001
PA0427^*a*^	oprM	Outer membrane efflux protein	1.52	<0.001
PA4596	esrC	Transcriptional regulator of efflux	1.08	<0.001
Siderophore-mediated iron uptake				
PA4513^*a*^	piuC	Inner membrane siderophore oxidoreductase	1.97	<0.001
PA4217	phzS	Flavin-containing monooxygenase	0.56	0.0022
PA4218	ampP/fptX	Pyochelin membrane transporter	1.84	<0.001
PA4219	ampO	Inner membrane protein	1.85	<0.001
PA4222	pchI	Pyochelin ABC transport protein	1.66	<0.001
PA4223	pchH	Pyochelin ABC transport protein	1.68	<0.001
PA4224	pchG	Pyochelin biosynthesis protein	2.01	<0.001
PA4225	pchF	Pyochelin biosynthesis protein	1.92	0.01
PA4229	pchC	Pyochelin biosynthesis protein	2.07	<0.001
PA4231	pchA	Pyochelin biosynthesis protein	1.33	<0.001
PA3465	yfiS	Enterobactin efflux transporter-like protein	0.53	0.0018
Ion transport and redox homeostasis				
PA0545		Ferric reductase	2.98	<0.001
PA3575^*a*^	yceJ	Cytochrome b561/ferric reductase	4.3	<0.001
PA3576		Periplasmic lipocalin-like protein	2.36	<0.001
PA3963		Probable iron transporter	2.54	<0.001
PA3963a		Readthrough variant of PA3963 with part of PA3964 disordered protein	5.41	<0.001
PA3795^*a*^		Cytoplasmic oxidoreductase	2.66	<0.001
PA2524^*a*^	czcS	Metal ion-sensing sensor histidine kinase	1.27	<0.001
PA0809	mtnH2	Divalent metal cation transporter	0.66	<0.001
BqsSR Fe(II)-sensing two-component system regulon				
PA2656	bqsS/carS	Iron-sensing sensor histidine kinase	3.13	<0.001
PA2657	bqsR/carR	Iron-sensing response regulator	3.9	<0.001
PA2658	bqsQ	BqsSR accessory protein	4.95	<0.001
PA2659	bqsP	BqsSR accessory protein	4.47	<0.001
PA0102	pSCA1	Beta-carbonic anhydrase	1.24	<0.001
PA0320	carO	Periplasmic substrate binding protein	7.4	<0.001
PA0321		Acetylpolyamine amidohydrolase	3.93	<0.001
PA0322		Polyamine/amino acid permease	1.17	<0.001
PA0327	carP	Inner membrane beta-propeller protein	4.61	<0.001
PA0328	aaaA	Arginine autotransporter	1.64	<0.001
Adhesins				
PA3059^*a*^	pelF	Pel glycosyltransferase	0.63	0.0033
PA3061^*a*^	pelD	Pel biosynthesis protein	1.07	<0.001
PA3063^*a*^	pelB	Pel biosynthesis protein	0.82	<0.001
PA4624	cdrB	Outer membrane adhesin export protein	0.60	<0.001
SOS Response				
PA0612	ptrB	Pyocin regulator	1.07	<0.001
PA0613		Pyocin protein	0.98	<0.001
PA0614		Pyocin protein	1.18	<0.001
PA0615		Pyocin protein	0.69	<0.001
PA0616		Pyocin protein	1.18	<0.001
PA0617		Pyocin protein	1.28	<0.001
PA0618		Pyocin protein	1.17	<0.001
PA0619		Pyocin protein	1.08	<0.001
PA0620		Pyocin protein	1.01	<0.001
PA0621		Pyocin protein	1.0	<0.001
PA0622		Pyocin protein	1.13	<0.001
PA0623		Pyocin protein	1.19	<0.001
PA0624		Pyocin protein	1.13	<0.001
PA0625		Pyocin protein	1.03	0.0012
PA0626		Pyocin protein	0.97	0.0018
PA0628		Pyocin protein	1.13	0.0036
PA0630		Pyocin protein	1.07	<0.001
PA0631		Pyocin protein	1.06	0.0064
PA0633		Pyocin protein	1.27	<0.001
PA0635		Pyocin protein	1.23	<0.001
PA0636		Pyocin protein	1.11	<0.001
PA0637		Pyocin protein	1.13	<0.001
PA0638		Pyocin protein	1.11	<0.001
PA0639		Pyocin protein	1.16	<0.001
PA0640		Pyocin protein	0.98	0.0029
PA0641		Pyocin protein	1.01	<0.001
PA0643		Pyocin protein	0.66	0.0005
PA0669	dnaE2	Damage-inducible polymerase	0.46	0.0015
PA0670		DNA repair protein	0.67	0.0022
PA0787		RecF-like DNA repair protein	0.40	<0.001
PA0807	ampDh3	Cell wall hydrolase	0.98	<0.001
PA0907	alpA	Autolysis protein	0.69	<0.001
PA0910	alpD	Autolysis protein	0.88	<0.001
PA0911	alpE	Autolysis protein	0.87	<0.001
DEGs downregulated in PAO1 *cpxS*^T163P^				
Cellular respiration and denitrification				
PA0509	nirN	Heme d (1) biosynthesis protein	−1.72	<0.001
PA0510	nirE	Heme d (1) biosynthesis protein	−1.56	0.0013
PA0511	nirJ	Heme d (1) biosynthesis protein	−0.92	0.0058
PA0520^*a*^	nirQ	Denitrification regulatory ATPase protein	−1.24	<0.001
PA0521	nirO	Cytochrome c oxidase subunit III	−1.24	0.0015
PA0522	nirP	Cytochrome c oxidase subunit IV	−1.68	<0.001
PA0523	norC	Nitric oxide reductase subunit C	−1.16	0.0026
PA0524	norB	Nitric oxide reductase subunit B	−1.12	0.0075
PA4129		NirA-associated oxidoreductase	−1.38	<0.001
PA4130	nirA	Nitrite reductase	−1.29	<0.001
PA4131	ccoG	Cytochrome *cbb_3_*-type oxidase assembly protein	−1.07	0.0058
PA4132	mpaR	Transcriptional regulator	−0.66	<0.001
PA4133	ccoN4	Cytochrome *cbb_3_*-type oxidase subunit	−0.86	<0.001
Fe-S cluster biogenesis				
PA1847	nfuA	Fe-S biogenesis protein	−0.85	<0.001
PA3811	hscB	Fe-S cofactor chaperone protein	−0.62	<0.001
PA3813	iscU	Fe-S assembly scaffold protein	−0.56	0.0021
PA3814	iscS	Fe-S biogenesis protein	−0.81	<0.001
PA3815	iscR	Transcriptional regulator	−0.66	0.0026
PA5275	cyaY	Fe-S biogenesis protein	−0.68	<0.001
Carbohydrate uptake				
PA3186	oprB	Outer membrane glucose porin	−1.22	<0.001
PA3187	gltK	Glucose transport protein	−1.44	<0.001
PA3188	gltG	Glucose transport protein	−1.21	<0.001
PA3189	gltF	Glucose transport protein	−1.34	<0.001
PA3190	gltB	Glucose permease	−1.74	<0.001
PA2291	oprB2	Outer membrane glucose porin	−0.34	0.0039
Motility and chemotaxis				
PA0395	pilT	Twitching motility protein	−0.32	<0.001
PA0413	chpA	Pil-Chp chemosensory protein	−0.51	<0.001
PA0414	chpB	Pil-Chp methylesterase	−0.50	<0.001

^*a*^ Indicate genes or operons predicted to be direct regulatory targets of CpxR.

^*b*^ Differential gene expression was determined by RNA-Seq analysis of a PAO1 *cpxS*^T163P^ mutant strain, which constitutively activates the Cpx response, and its wild-type parental strain. Transcriptional profiling was performed on bacteria grown to mid-exponential phase in unstressed LB media. Log_2_ fold change (FC) and adjusted *P*-value (*P*_adj_) from DESeq2 output are displayed (83). A cutoff of (*P*_adj_) < 0.01 was imposed when evaluating differential gene expression.

Many of the genes significantly upregulated in PAO1 *cpxS*^T163^ are related to iron acquisition. Iron is essential for bacterial growth, but its abundance is often limited during infection due to host competition and limited solubility of oxidized iron (Fe(III)) in aerobic environments. *P. aeruginosa* scavenges extracellular iron by producing a variety of secreted high-affinity Fe(III)-binding siderophores. Genes involved in the production and assimilation of the siderophore pyochelin were upregulated, as well as a variety of redox enzymes involved in reducing Fe(III) to Fe(II), which has improved bioavailability and can be readily assimilated by the cell. Cpx-activating mutations have also been associated with increased secretion of the siderophore pyoverdine via the MuxABC-OpmB pump, suggesting a potentially multifaceted role of Cpx in iron regulation (44, 86). Of the genes predicted as CpxR direct targets that did not exhibit significant differential expression in PAO1 *cpxS*^T163P^, we note that several (*pirA*/*PA0931*, *PA3268*, *fpvB*/*PA4168*, *piuA*/*PA4514*) encode cell-surface siderophore receptors, which often require the presence of their ferrisiderophore cargo for full expression, a feature likely limited under our iron-rich conditions (87). A previous transcriptomic analysis of the Cpx response in *Vibrio cholerae* El Tor C6706 similarly revealed upregulation of iron acquisition factors, including several orthologous genes to those identified here (Table S3) (64).

PAO1 *cpxS*^T163P^ also exhibits elevated expression of genes encoding the metal ion-induced two-component systems CzcRS and BqsRS. *CzcRS* is a predicted target of CpxR and regulates cellular efflux and outer membrane permeability in response to high concentrations of various heavy metal ions (88, 89). The BqsRS (CarRS) system senses reduced iron (Fe(II)) (90, 91); *bqsRS* and its regulon were among the highest upregulated genes identified in this study and were similarly upregulated by Cpx in *Vibrio cholerae* (63). Given the abundance of iron acquisition and redox factors upregulated in PAO1 *cpxS*^T163P^, we hypothesize that elevated Cpx activity increases the cellular concentration of bioavailable Fe(II) iron, leading to induction of BqsRS. Elevated cellular iron in PAO1 *cpxS*^T163P^ may also account for the broad upregulation of genes associated with the cellular response to oxidative damage, including a large stress-inducible R2/F2-type pyocin gene cluster, as free iron and ferrisiderophores generate damaging reactive oxygen species through Fenton/Haber-Weiss chemistry (92–95).

We observed a significant reduction in the expression of the denitrification regulatory gene *nirQ*, a predicted CpxR target, indicating that PAO1 CpxR can exert both stimulatory and inhibitory effects on target promoters. We observed that the predicted CpxR-binding site upstream of the *nirQ* gene overlaps a known binding site for the global denitrification regulator Anr, which is essential for expression of both diverging *nirQ* and *nirS* genes, the latter encoding the precursor for nitrite reductase (96). Other denitrification-related genes, particularly those involved in the production of heme-containing and iron-sulfur enzymatic cofactors, were downregulated in *cpxS*^T163P^. Suppression of these functions may serve as a protective mechanism for *P. aeruginosa* under envelope stress, where these chemically labile cofactors can damage cell structures through Fenton chemistry (95, 97, 98).

### The Cpx system is activated by surface attachment and is not linked to c-di-GMP-based surface sensing

The *E. coli* Cpx system is generally considered to be induced upon bacterial surface contact, though this phenomenon significantly varies based on experimental conditions such as surface type (22, 85, 99, 100). In the current model, *E. coli* Cpx senses surface adhesion through a mechanism involving the CpxA periplasmic sensor domain and outer membrane-localized proteins, including the lipoprotein sensor NlpE and the outer membrane β-barrel protein OmpA (22, 100, 101). *P. aeruginosa* lacks an obvious NlpE homolog, and whether the Cpx system is induced by surface attachment is unclear. To investigate, we assessed the activity of a P*_cpxP_*::mGL fluorescent reporter among cells from planktonic and surface-attached populations. A similar approach was previously used in our lab to monitor a temporal increase in c-di-GMP signaling following *P. aeruginosa* surface contact, in which the activity of a fluorescent c-di-GMP-sensitive promoter (P*_cdrA_*) was quantified in surface-attached cells at a liquid:solid interface (8).

**Fig 5:**
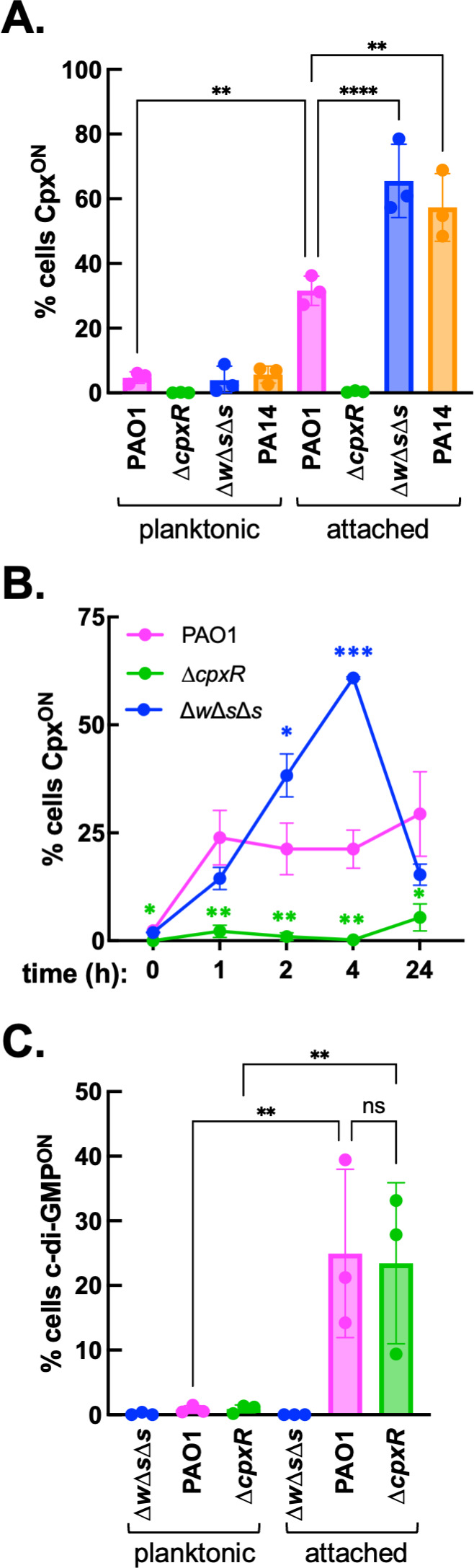
The Cpx system is induced in surface-attached *P. aeruginosa* independent of c-di-GMP. (**A**) Single-cell activity of a P*_cpxP_*::mGreenLantern/P*_rpoD_*::mScarlet fluorescent reporter plasmid was quantified by microscopic imaging of fixed *P. aeruginosa*. Bacteria were grown to mid-exponential phase in LB for 3 h (Abs600 ~ 0.5) and either fixed immediately (planktonic cell fraction) or exposed to a LB 1.5% agarose surface for an additional 4 h, after which the attached cell fraction was harvested. Wild-type PAO1 and isogenic mutants PAO1 ∆*cpxR* and PAO1 ∆*wspR*∆*sadC*∆*siaD* (∆*w*∆*s*∆*s*), as well as model isolate PA14, were analyzed. Cells were designated Cpx^ON^ when normalized P*_cpxP_* reporter activity exceeded 5× an experimentally determined arbitrary threshold, defined by the mean normalized reporter activity from the planktonic PAO1 ∆*cpxR* population. Graphed are means (± SD) of the %Cpx^ON^ cells per condition, with asterisks denoting statistical significance based on ordinary one-way ANOVA with multiple comparisons test; **P < 0.01, ****P < 0.0001. (**B**) Time-course analysis of P*_cpxP_* reporter activity in PAO1 and derivative mutant strains following exposure to a liquid:agarose interface, with 0 h representing planktonic cells from the initial inoculum. Asterisks denote statistical significance based on pairwise t-test between PAO1 and mutant strains at the indicated time points; *P < 0.05, **P < 0.01, ***P < 0.001. (**C**) PAO1 and derivative mutants expressing a P*_cdrA_*::mGreenLantern/P*_rpoD_*::CFP fluorescent reporter plasmid were assessed for surface-induced activity of the c-di-GMP sensitive promoter P*_cdrA_* as described in (**A**), normalizing P*_cdrA_* to P*_rpoD_*. Cells were considered P_cdrA_/c-di-GMP^ON^ when normalized PcdrA activity exceeded 5× the mean value from the planktonic PAO1 ∆*w*∆*s*∆*s* population. Graphed are means (± SD) of the %c-di-GMP^ON^ cells per condition, asterisks based on one-way ANOVA with multiple comparisons test; **P < 0.01, “ns” no significant difference. All data are pooled from three independent experiments, with >100 cells assessed per biological replicate.

We found that P*_cpxP_*::mGL fluorescent signal was substantially elevated in surface-attached *P. aeruginosa* (Fig. 5A); strong single-cell heterogeneity in reporter activity was observed, particularly among attached cells, with many individuals exhibiting at least a fivefold increase in normalized P*_cpxP_*::mGL activity relative to a ∆*cpxR*-negative control population. For strain PAO1, we found that around a quarter of the attached cell population showed high Cpx activity (Cpx^ON^) after 4 h surface attachment. Another widely studied *P. aeruginosa* isolate, PA14, exhibited a stronger Cpx surface response, with >60% attached cells Cpx^ON^. A time-course analysis of P*_cpxP_*::mGL reporter activity in PAO1 further indicated that the Cpx response is elicited rapidly upon initial surface contact; PAO1 reporter activity rose to ~25% cells Cpx^ON^ 1 h post-surface attachment and remained constant at later time points (Fig. 5B). We similarly assessed Cpx surface signaling among various PAO1 ∆*cpx* gene deletion mutants and found that both the core two-component system CpxRS and adaptor CpxM are required for PAO1 to mount a full Cpx surface response (Fig. S12A). A functional Cpx signaling system was not required for effective PAO1 colonization of surfaces at the liquid:solid interface (Fig. S12B), suggesting that the Cpx system is important post-attachment.

To determine whether the Cpx surface response was affected by c-di-GMP levels, we similarly tested reporter activity of a PAO1 ∆*wspR*∆*sadC*∆*siaD* (∆*w*∆*s*∆*s*) mutant lacking the three major diguanylate cyclases responsible for intracellular c-di-GMP accumulation in response to surface contact (8, 102). This strain displays an overall reduction in initial (1–4 h) surface colonization (Fig. S13), consistent with previous observations that *P. aeruginosa* with low c-di-GMP levels typically exhibit increased cellular motility and fail to remain stably adhered to a surface (72). PAO1 ∆*w*∆*s*∆*s* exhibited a strong Cpx surface response similar to PA14 (>60% Cpx^ON^), significantly exceeding Cpx reporter levels observed in the PAO1 parental strain (Fig. 5A). Time-course analysis of P*_cpxP_* reporter activity further revealed that PAO1 ∆*w*∆*s*∆*s* exhibited a transient increase in Cpx signaling 2–4 h post-attachment, which returned to PAO1 levels by 24 h (Fig. 5B). While the molecular basis of this phenotype is subject to ongoing investigation, these data indicate that Cpx surface sensing does not involve the cyclases linked to the c-di-GMP surface response, but may be promoted during the transient, unstable surface interactions characteristic of c-di-GMP-defective *P. aeruginosa*. Correspondingly, surface-induced activity of the c-di-GMP reporter P*_cdrA_*::mGL did not significantly differ between a PAO1 ∆*cpxR* mutant and its parental strain (Fig. 5C), similarly suggesting that Cpx signaling does not impact the c-di-GMP surface response. Together, these findings suggest that the Cpx and c-di-GMP surface responses function independently.

## DISCUSSION

Relative to the well-characterized landscape of ESRs in Enterobacteriaceae, envelope stress signaling in *P. aeruginosa* is less understood. In the *E. coli* paradigm system, the cell envelope is surveilled by a network of ESRs, including σ^E^, Cpx, Bae, Psp, and Rcs (12). Based on sequence homology, *P. aeruginosa* PAO1 possesses complete orthologs of only the Cpx and σ^E^ (AlgU) systems. The homologous σ^E^ and AlgU extracytoplasmic sigma factors similarly regulate expression of genes involved in assembly of the cell wall, LPS, and membrane proteins, many of unknown function (103–105). Outside of this core set of envelope-related functions, the full σ^E^ regulon appears to vary within the *E. coli* species group and across bacterial species (106, 107), including regulation of diverse virulence traits such as the biofilm exopolysaccharide alginate, an important factor in chronic *P. aeruginosa* pulmonary infections (108). In this study, we found that the Cpx system of *P. aeruginosa* also exhibits significant divergence from its *E. coli* ortholog, including novel Pseudomonas-specific Cpx adaptor proteins and evident differences in the Cpx regulon. The dissimilar envelope stress responses in *E. coli* and *P. aeruginosa* may reflect the significant ecological and pathogenic differences of these bacteria (an obligate animal commensal vs. an environmentally acquired opportunistic pathogen, respectively).

These apparent species-level differences highlight a need for further *in situ* investigation of Pseudomonas ESR signaling networks. The exceptionally large repertoire of two-component systems in *P. aeruginosa* (>60 vs. ~30 in *E. coli* K-12) (109) potentially contains various novel ESRs, with several systems involved in envelope stress tolerance already identified (i.e., PhoPQ, PmrAB, AmgRS, DsbRS) (110–115). Of these, AmgRS and DsbRS have been shown to directly regulate envelope homeostasis factors (i.e., *htpX*, *dsb*) that are typically regulated by the Cpx system in *E. coli*, but absent from the Cpx regulon in *P. aeruginosa*. Compartmentalization of the envelope stress response across a multiplicity of sensory systems may enable *P. aeruginosa* to fine-tune its response to specific stress-related stimuli, and functional redundancy among systems may permit evolution of specialized regulators linking cell ESR signaling to other biological processes, including virulence and biofilm regulation. Diversification of two-component signaling with adaptor proteins may further expand the *P. aeruginosa* ESR network. The incorporation of Pseudomonadaceae-specific accessory genes into broadly conserved two-component systems (i.e., BqsRS, CbrAB) has been described previously (90, 116). In this study, we investigated the function of two novel Cpx adaptor genes, *cpxM* (*PA3203*) and *cpxH* (*PA3207*), finding that *cpxM* is required for full Cpx signaling in PAO1, including Cpx induction in response to *P. aeruginosa* surface contact. The predicted periplasmic domain of the CpxM protein may facilitate interaction(s) with Cpx-inducing ligands, and/or this adaptor may potentiate CpxS activation through interactions within the inner membrane. While the histidine phosphotransferase-like protein CpxH is nonessential for the Cpx response, this factor may enhance Cpx phosphorelay activity or facilitate regulatory crosstalk with other systems. As this protein is present only in *P. aeruginosa*, it is likely a more recent acquisition of the Cpx system and represents an intermediate state in the evolution of a novel two-component signaling mechanism. Further investigations into the cellular functions of CpxM and CpxH are currently ongoing.

Our work substantiates that the *P. aeruginosa* Cpx system, as in other species, is induced by cell envelope stress. By targeting specific envelope functions with CRISPRi, we furthermore discovered that OMP dysbiosis (i.e., due to *bamA* transcriptional arrest by CRISPRi) is a major inducing cue of the PAO1 Cpx system. When the Bam function is disrupted, uninserted β-barrel OMPs likely accumulate in the periplasm, where they may activate Cpx due to misfolding or erroneous insertion in the inner membrane (117, 118). The *E. coli* Cpx system is similarly induced under conditions where OMP assembly is dysregulated, though the underlying mechanism(s) are not fully understood (119, 120). The *P. aeruginosa* Cpx system may be regulated by a dedicated outer membrane protein sensor, similar to interactions previously described between *E. coli* CpxA and the porin OmpA (22, 121). Our observation that the *P. aeruginosa* CpxP adaptor protein bears strong similarity to *E. coli* Spy, a periplasmic chaperone known to bind unfolded outer membrane proteins, may further suggest that *P. aeruginosa* Cpx signaling involves an unknown outer membrane protein factor.

The *P. aeruginosa* Cpx system was also stimulated by CRISPRi targeting of *uppS*, which depletes cellular availability of the inner membrane carrier lipid C55-PP. The molecular basis of this response is unclear, particularly considering that direct inhibition of cell envelope biogenesis processes requiring carrier lipids, such as cell wall peptidoglycan and LPS production, did not induce Cpx signaling to the same degree. Pleiotropic effects of *uppS* deletion, impacting the stability of multiple envelope compartments, may contribute to Cpx activation. We note that the sensitivity of our reporter is potentially limited to detecting large changes in Cpx signaling. We observed significant Cpx reporter induction in response to depletion of *bamA*, consistent with a recent RNA-Seq analysis using the same *bamA* sgRNA in PAO1, which reported a ~20-fold upregulation in *cpxP* transcript levels (75); however, that study also reported ~6-fold upregulation of *cpxP* under *lptD* depletion, which did not significantly impact activity of our reporter. Another prior study reported that *cpxP* is upregulated under inhibition of lipoprotein trafficking in PAO1 (122), in contrast to our findings that *lolA* and *lolC* depletion do not elicit Cpx reporter activity, though substantial differences in experimental approach may account for this discrepancy (123, 124). Further investigation is therefore necessary to determine the scope and nature of Cpx-inducing stress stimuli in *P. aeruginosa*.

Consistent with the *E. coli* literature, we also observed that surface attachment stimulates the Cpx system in *P. aeruginosa* through a mechanism independent of the global biofilm regulator c-di-GMP. The magnitude of the Cpx surface response differed between isolates PAO1 and PA14, which are often used as representatives of the two major phylogenetic clades of *P. aeruginosa* (125) and exhibit distinct patterns of surface attachment and biofilm formation (126). PAO1 cells rapidly form stable associations with a surface, often “laying down” along the cell body, accompanied by an early accumulation of c-di-GMP (i.e., induction of a c-di-GMP-sensitive fluorescent reporter after 4 h surface attachment) (8, 72). Meanwhile, PA14 favors initial surface adhesion at the cell pole and exhibits a relatively prolonged period of “sampling” the surface through transient, reversible attachment (127). This behavioral pattern slows the initial progression of surface colonization, with c-di-GMP not significantly accumulating in PA14 until ~24 h (128). Overall, our findings suggest that the Cpx response is rapidly induced upon initial surface contact and predominates in the early stages of *P. aeruginosa* surface interaction. The heightened Cpx surface response observed among strains lacking an early c-di-GMP surface response (PA14, PAO1 ∆*w*∆*s*∆*s*) may therefore accumulate through multiple successive “surface-sampling” contact events, as cells engage in transient attachment events before committing to biofilm formation.

The consequences of Cpx surface signaling on biofilm formation remain to be fully explored. Our data suggest that Cpx may regulate the production of the cell-associated exopolysaccharide Pel, providing an additional, c-di-GMP-independent link between surface sensing and biofilm matrix production. Unlike c-di-GMP, however, Cpx does not appear to broadly suppress the expression of genes involved in motility (i.e., flagella, pili) in *P. aeruginosa*. An alternative mechanism for surface-engaged cells to initiate exopolysaccharide production, while retaining some level of cellular motility, may enable a bacterial population to effectively distribute and arrange itself as it colonizes a new surface to maximize overall biofilm fitness. Other prospective Cpx-regulated processes identified in this study, such as iron acquisition, are also associated with *P. aeruginosa* biofilm formation. Given that sufficient concentrations of iron are required for surface-associated *P. aeruginosa* cells to commit to biofilm formation (129, 130), Cpx activation may “prime” transient or peripherally attached cells to increase the uptake of this key nutrient. This initial surface response may therefore lay the foundation for the progression of biofilm formation once other environmental criteria for biofilm initiation, such as adequate levels of environmental iron, are met. Other Cpx-regulated factors, such as those involved in redox homeostasis, may enable cells to acclimate to the unique physiological conditions of the surface, which can differ significantly from aqueous environments (9, 131). Future studies will investigate the effects of the Cpx surface response on individual cell fate during surface colonization, as well as the coordination of Cpx with other surface-sensing systems, to evaluate this model.

## MATERIALS AND METHODS

### Strains and growth conditions

*P. aeruginosa* and *E. coli* strains were stored at −80°C in 25% glycerol and routinely streaked onto Lennox LB medium 1.5% agar plates. Plates were incubated either overnight at 37°C or (for *P. aeruginosa*) 2 days at room temperature. Bacteria were inoculated aseptically into 2 mL sterile LB broth in borosilicate glass test tubes and grown overnight (~18–20 h) at 37°C with 225 RPM agitation. When necessary, LB was supplemented with gentamicin to concentrations of 30 µg/mL for chromosomal markers and 100 µg/mL for plasmid-based markers in *P. aeruginosa*, and 10 µg/mL for *E. coli*.

### Genetic analysis of PAO1 Cpx

Gene deletion mutants were constructed in PAO1 by allelic exchange with the nonreplicative vector pEX18 as previously described (132, 133). Mutant genotypes were confirmed by PCR and Sanger sequencing. For complementation, wild-type gene alleles were PCR amplified from PAO1 genomic DNA (gDNA) using primers containing an artificial strong bacterial ribosome-binding (RBS) sequence to enhance transgene expression and cloned into an L-arabinose-inducible expression cassette (*araBAD*) in the replicative vector pJN105 (Gm^R^) using Gibson assembly (134). Plasmids were transformed into competent PAO1 strains by electroporation (135). 187 bp upstream of the *cpxP* (*PA3205*) start codon was PCR amplified from PAO1 gDNA, using primers to introduce an artificial strong bacterial RBS, and Gibson cloned upstream of a lacZ reporter gene into a linearized nonreplicative vector pUC18-miniTn7T (Gm^R^) (136). Tn7 integrated transformants were cured of the selective marker by FRT recombination with the plasmid pFLP (Cb^R^), which was subsequently cured by *sacB* counterselection (137). Overnight bacterial cultures were subcultured in LB containing gentamicin (for pJN105 plasmid retention) and 0.1% L-arabinose, as well as 2% ethanol to induce cellular stress where indicated, and grown 3 h at 37°C with 225 RPM agitation. 50 µL aliquots were transferred to a 96-well microplate, and turbidity (Abs_600_) was measured in a CLAIROstar PLUS microplate reader (BMG Labtech). Cells were lysed by a 1:2 dilution in B-PER II protein extraction reagent (Thermo Scientific). P_cpxP_::*lacZ* reporter activity was quantified by a standard β-galactosidase assay (138).

### Fluorescence microscopy of Cpx reporter activity

We adapted a pBBR1-MCS5-based (Gm^R^) reporter plasmid described previously (8, 139), using Gibson assembly to fuse the *cpxP* gene promoter (187 bp) to a green fluorescent protein mGreenLantern (140, 141). This plasmid also contained a P*_rpoD_*::mScarlet-I red fluorescent protein housekeeping reporter for normalization (6, 142). Short half-life (ASV) variants of fluorescent proteins were utilized (8, 141), and all promoter fusions contained an artificial RBS to enhance reporter expression. This construct was used to report Cpx activity in both PAO1 and PA14, as the *cpxP* promoter region is > 99% identical between strains. Reporter activity was quantified by imaging cells with a Nikon Ti2 Eclipse widefield inverted microscope with a 100× objective oil-immersion lens, using phase contrast and fluorescence filters for GFP (Ex 466/40, Em 525/50) and TRITC (Ex 554/23, Em 609/54). Light intensity and exposure time were adjusted as needed to optimize visibility. Prior to imaging, cells were fixed in 2.5% paraformaldehyde in 1× phosphate-buffered saline (PBS), incubated >15 min at 37°C with agitation, and stored at 4°C for <48 h. 1–5 µL of fixed cells were spotted onto a glass coverslip and overlaid with a pad of 1% low melting point agarose in 1× PBS. At least 100 individual cells were imaged for all biological replicates. Images were processed using the MicrobeJ plugin for the Fiji distribution of ImageJ (143), with pre-processing to detect bacterial cells performed as described previously (8). Background-extracted mean fluorescence intensity values for individual cells were analyzed with Microsoft Excel or R. GraphPad Prism v10.3.0 was used for graphing and statistical analysis.

### Cpx elicitor screen

Bacteria expressing the pBBR1 P*_cpxP_*::mGreenLantern/P*_rpoD_*::mScarlet-I reporter were grown overnight in LB with gentamicin selection and subcultured 1:100 in fresh LB supplemented with the indicated chemical stressors. After 4 h growth at 37°C with 225 RPM agitation, culture turbidity (Abs_600_) was measured by Genesys20 tube spectrophotometer, and cells were fixed and imaged by Nikon Ti2 Eclipse (10% light intensity, with 200 ms exposure for the GFP channel and 400 ms for the TRITC channel). We filtered out non-fluorescing cells due to plasmid loss, excluding all cells with <800 arbitrary fluorescence units (AFUs) in the P*_rpoD_*::mScarlet channel from further analysis. Bacteria were designated as Cpx^ON^ when normalized P*_cpxP_*::mGreenLantern/P*_rpoD_*::mScarlet fluorescent signal exceeded 2× the mean value from a control population grown in unstressed LB (72).

### CRISPRi

Our approach is based on that described previously (75). An *araBAD*::*sp*dcas9 cassette was PCR amplified from an integrated *att*B::*araBAD::sp*dcas9 construct in PAO1, gifted by the Mekalanos lab. The *araBAD*::*sp*dcas9 cassette was amplified from gDNA by PCR and cloned into the nonreplicative Tn7 integration plasmid, pUC18-miniTn7T (GmR), by Gibson assembly. A cassette containing both a 25-nucleotide sgRNA and a gRNA scaffold was expressed under SpeI constitutive promoter control on a pPSV37 plasmid; vectors containing *bamA*, *lptD*, and *secY* sgRNAs were also received from the Mekalanos Lab. All other sgRNA vectors were generated in this study by PCR amplification of the pPSV37 backbone with Gibson overhangs introducing sgRNA sequences, which were then DpnI treated and re-circularized by Gibson ligation. For validation of CRISPRi target inhibition, bacterial growth (Abs_600_) was assessed by CLAIROstar PLUS microplate reader (BMG Labtech), using overnight bacterial cultures diluted 1:100 into fresh LB media with gentamicin and 0.2% L-arabinose. To confirm inhibition of the nonessential gene *dsbA* by phenotypic screening, 5 µL of bacteria was spotted onto Vogel-Bonner Minimal Medium 1.5% agar plates containing 80 µg/mL Congo Red, 30 µg/mL Brilliant Blue, 100 µg/mL gentamicin, and 1% L-arabinose (144). To confirm inhibition of *algU*, the *mucA22* mutation was introduced into PAO1 Tn7::*araBAD::sp*dcas9 by allelic exchange, conferring a mucoid phenotype (145, 146). Strains were plated onto Pseudomonas Isolation Agar with gentamicin and 1% L-arabinose to confirm that sgRNA targeting of *algU* reversed the mucoid phenotype. To assess the impact of CRISPRi on Cpx activity, sgRNA expression cassettes were Gibson cloned into pBBR1 P*_cpxP_*::mGreenLantern/P*_rpoD_*::mScarlet-I. Bacterial cultures were diluted 1:100 in fresh LB with gentamicin and 0.2% L-arabinose and grown 4 h at 37°C with 225 RPM agitation. Cells were fixed and imaged by a Nikon Ti2 Eclipse as described above, using 20% light source intensity with 200 ms exposure for both GFP and TRITC channels. Due to plasmid loss among cells expressing toxic transgenes, we excluded non-fluorescing cells (<100 AFUs in the P*_rpoD_*::mScarlet channel) from further analysis. Bacteria were designated as Cpx^ON^ when the normalized P*_cpxP_*::mGreenLantern/P*_rpoD_*::mScarlet fluorescence intensity exceeded 2× the mean value from a control PAO1 population, which contained the Tn7::*araBAD::sp*dcas9 cassette and fluorescent reporter plasmid with no sgRNA.

### CpxR-binding site prediction

We queried the PAO1 genome (NC_002516.2, Pseudomonas Genome Database) for the CpxR consensus sequence (5′-GTAAA-(N)_4-8_-GTAAA-3′) (39), and a near-perfect 5′-GTAAA-(N)_4-8_-GTAA-3′ motif previously identified in Cpx-regulated promoters in *E. coli* (23). We included two additional CpxR motifs (5′-GTCAA-(N)_4-8_-GTAAA-3′ and 5′-GTAAA-(N)_4-8_-GTCAA-3′) identified through exponential enrichment (HT-SELEX) of DNA binding interactions by PAO1 CpxR *in vitro* (79). Of the 40 total sites identified by this method, 22 potential CpxR-regulated gene targets were selected based on the presence of at least one CpxR-binding motif within 500 bp upstream of the translation initiation codon. We additionally surveyed potential CpxR targets identified by a DAP-Seq analysis of the genomic binding profiles of >50 response regulators in PAO1 (82). To limit analysis to high-likelihood targets, we considered only binding sites that showed 10× or greater enrichment when precipitated with CpxR relative to a negative DAP-Seq control. Of 28 potential CpxR targets, 15 had already been identified by our analysis of CpxR consensus motifs. For the remaining 13 targets predicted by Trouillon et al., we estimated potential CpxR promoter-binding motifs based on the location of DAP-Seq peak summit positions, identified by the original authors using MASC2 followed by BEDtools analysis (147, 148). These sites typically contained one CpxR direct-repeat sequence (5′-GTAAA-3′) or similar motif. In cases where predicted CpxR sites fell within an intergenic region with two potential gene targets, we favored the gene in which the binding site was positioned approximately 50–200 bp upstream of the start codon, as binding site location is a strong predictor of Cpx regulation in *E. coli* (26).

### RNA-Seq

Bacterial overnight cultures were subcultured 1:100 in LB and grown to an optical density (Abs_600_) of 0.52 ± 0.01 as measured by a Genesys20 test tube spectrophotometer. Cultures were normalized to Abs_600_ = 0.1. 1 mL (~1 × 10^8^ cells) was sedimented by centrifugation for 5 min at 17,000 × *g* and pellets were flash frozen in liquid nitrogen. Standard RNA preparation, including rRNA depletion and Illumina library preparation, was performed by Azenta Life Sciences, and paired-end 150 bp sequencing was performed on an Illumina HiSeq 3000. Trimmomatic was used to trim TruSeq3 adapter sequences and to filter low-quality reads (149). Reads were aligned to the reference PAO1 genome (NC_002516.2) with STAR (150, 151). Htseq-count was used to quantify reads overlapping genomic features as defined by a GFF3 annotation file obtained from Pseudomonas Genome DB (152). Differential gene expression (DGE) analysis was performed with DESeq2 (83). Operon prediction was performed on trimmed reads using Rockhopper (53).

### qRT-PCR validation of DGE

PAO1 and PAO1 *cpxS*^T163P^ were grown to mid-exponential phase as described above for RNASeq sample preparation. 2 mL culture was pelleted by centrifugation and flash frozen in liquid nitrogen. Pellets were resuspended in 500 µL TRIzol (Invitrogen) and extracted with 100 µL chloroform. Samples were centrifuged at 17,000 × *g* for 15 min at 4°C to separate phases, and the aqueous phase was combined with an equal volume of ice-cold 100% ethanol. RNA was purified by the RNeasy Mini column-based kit (Qiagen). Nucleic acid was eluted in DEPC-treated ddH_2_O, and contaminating gDNA was removed using a TURBO DNA-free kit (ThermoFisher). Purified RNA was quantified by Qubit RNA HS assay using a Qubit 3 fluorometer (Invitrogen by ThermoFisher). Samples were normalized to 3 µg total RNA in DEPC-treated ddH_2_O prior to cDNA synthesis with an AffinityScript QPCR cDNA Synthesis kit (Agilent). qRT-PCR was performed using the SsoAdvanced Universal SYBR Green Supermix (BioRad) and a BioRad CFX384 thermocycler. DGE was calculated by the 2^−∆∆Ct^ formula (153).

### Cpx surface sensing assay

*P. aeruginosa* with pBBR1 P*_cpxP_*::mGreenLantern/P*_rpoD_*::mScarlet-I was diluted 1:100 from overnight cultures grown at 37°C in LB broth for 3 h. 1 mL culture aliquots (~ 0.5 × 10^8^ CFUs) were sedimented by centrifugation for 10 min at 10,000 × *g*, and either fixed immediately in 2.5% paraformaldehyde in 1× PBS (planktonic cell fraction) or transferred to a sterile LB 1.5% agarose surface overlaid with 1 mL ddH_2_O in a 24-well microtiter plate. Plates were incubated statically at 37°C for 4 h. The supernatant was then decanted, and the agarose surface gently washed with 1 mL 1× PBS. The remaining surface-attached cell fraction was then resuspended in 200 µL of 2.5% paraformaldehyde by shaking for 20 min at 37°C. Cells were either imaged immediately or stored at 4°C for <48 h. Fluorescence microscopy was performed with a Nikon Ti2 Eclipse as described above, with exposure time set to 200 ms and Sola light source intensity set to 25% (GFP) and 10% (TRITC). Single-cell fluorescence intensity was quantified with MicrobeJ as previously detailed. Cells with P*_rpoD_*::mScarlet mean fluorescence intensity <100 AFU were omitted from further analysis; at least 200 individual cells were analyzed per biological replicate. Reporter activity was quantified by normalizing P*_cpxP_*::mGreenLantern to P*_rpoD_*::mScarlet fluorescence intensity values for each cell. Activity of a plasmid-based c-di-GMP responsive fluorescent reporter (pBBR1 P*_cdrA_*::mGreenLantern/P*_rpoD_*::CFP) was assessed by the same methods, using P_rpoD_::CFP signal for normalization (8). Both Sola (GFP) and Xylis (CFP) light sources were set to 20% intensity for experiments using this reporter. For each cell, the fold change in reporter activity was calculated relative to the mean value of an experimentally defined negative control population (reporter^OFF^), with cells exhibiting a >5× fold change designated reporter^ON^. For Cpx reporter assays, the planktonic cell population of PAO1 ∆*cpxR* was used to represent the reporter^OFF^ condition; for c-di-GMP reporter assays, the planktonic population of a c-di-GMP-deficient strain, PAO1 ∆*wspR*∆*sadC*∆*siaB*, defined the reporter^OFF^ condition.

### Surface attachment assay

PAO1 and derivative mutant strains carrying the pBBR1 P*_cpxP_*::mGreenLantern/P*_rpoD_*::mScarlet-I reporter plasmid were subcultured (1:100) from overnight cultures into fresh LB broth and grown 3 h at 37°C. 1 mL exponential-phase culture was combined with 1 mL ddH_2_O in a 12-well polystyrene microplate, and 22 × 22 mm glass coverslips (VWR) were submerged vertically in wells. Plates were incubated statically at 37°C, and coverslips were removed at the indicated times and submerged in a reservoir of 0.5% crystal violet stain in ddH_2_O for 15 min. Coverslips were washed thrice in ddH_2_O to remove residual staining and destained in 0.5 mL 30% acetic acid solution. Attachment was quantified by measuring absorbance at 562 nm of destained solution in a CLAIROstar PLUS microplate reader.

## Data Availability

RNA-Seq data were deposited in the NCBI Sequence Read Archive under accession no. PRJNA1238893. All study data are included in this article, with raw/unprocessed data and images available by request.

## SUPPLEMENTAL MATERIAL

Supplemental Figures mbio.02726-25-s0001.docx Fig. S1 to S13.

Supplemental Tables mbio.02726-25-s0002.xlsx Tables S1 to S5.

ASM does not own the copyrights to Supplemental Material that may be linked to, or accessed through, an article. The authors have granted ASM a non-exclusive, world-wide license to publish the Supplemental Material files. Please contact the corresponding author directly for reuse.

## References

[1] Donlan RM. 2001. Biofilm formation: a clinically relevant microbiological process. Clin Infect Dis 33:1387–1392. doi:10.1086/322972 11565080

[2] Costerton JW, Stewart PS, Greenberg EP. 1999. Bacterial biofilms: a common cause of persistent infections. Science 284:1318–1322. doi:10.1126/science.284.5418.1318 10334980

[3] Laventie BJ, Jenal U. 2020. Surface sensing and adaptation in bacteria. Annu Rev Microbiol 74:735–760. doi:10.1146/annurev-micro-012120-063427 32905753

[4] Schniederberend M, Williams JF, Shine E, Shen C, Jain R, Emonet T, Kazmierczak BI. 2019. Modulation of flagellar rotation in surface-attached bacteria: a pathway for rapid surface-sensing after flagellar attachment. PLoS Pathog 15:e1008149. doi:10.1371/journal.ppat.1008149 31682637 PMC6855561

[5] Luo Y, Zhao K, Baker AE, Kuchma SL, Coggan KA, Wolfgang MC, Wong GCL, O’Toole GA. 2015. A hierarchical cascade of second messengers regulates Pseudomonas aeruginosa surface behaviors. mBio 6:e02456-14. doi:10.1128/mBio.02456-14 25626906 PMC4324313

[6] Persat A, Inclan YF, Engel JN, Stone HA, Gitai Z. 2015. Type IV pili mechanochemically regulate virulence factors in Pseudomonas aeruginosa. Proc Natl Acad Sci USA 112:7563–7568. doi:10.1073/pnas.1502025112 26041805 PMC4475988

[7] O’Neal L, Baraquet C, Suo Z, Dreifus JE, Peng Y, Raivio TL, Wozniak DJ, Harwood CS, Parsek MR. 2022. The Wsp system of Pseudomonas aeruginosa links surface sensing and cell envelope stress. Proc Natl Acad Sci USA 119:e2117633119. doi:10.1073/pnas.2117633119 35476526 PMC9170161

[8] Zheng X, Gomez-Rivas EJ, Lamont SI, Daneshjoo K, Shieh A, Wozniak DJ, Parsek MR. 2024. The surface interface and swimming motility influence surface-sensing responses in Pseudomonas aeruginosa. Proc Natl Acad Sci USA 121:e2411981121. doi:10.1073/pnas.2411981121 39284057 PMC11441478

[9] Kimkes TEP, Heinemann M. 2020. How bacteria recognise and respond to surface contact. FEMS Microbiol Rev 44:106–122. doi:10.1093/femsre/fuz029 31769807 PMC7053574

[10] Chawla R, Gupta R, Lele TP, Lele PP. 2020. A Skeptic’s guide to bacterial mechanosensing. J Mol Biol 432:523–533. doi:10.1016/j.jmb.2019.09.004 31629771 PMC7002054

[11] Chevalier S, Bouffartigues E, Tortuel D, David A, Tahrioui A, Labbé C, Barreau M, Tareau AS, Louis M, Lesouhaitier O, Cornelis P. 2022. Cell envelope stress response in Pseudomonas aeruginosa. Adv Exp Med Biol 1386:147–184. doi:10.1007/978-3-031-08491-1_6 36258072

[12] Mitchell AM, Silhavy TJ. 2019. Envelope stress responses: balancing damage repair and toxicity. Nat Rev Microbiol 17:417–428. doi:10.1038/s41579-019-0199-0 31150012 PMC6596312

[13] Mecsas J, Rouviere PE, Erickson JW, Donohue TJ, Gross CA. 1993. The activity of sigma E, an Escherichia coli heat-inducible sigma-factor, is modulated by expression of outer membrane proteins. Genes Dev 7:2618–2628. doi:10.1101/gad.7.12b.2618 8276244

[14] Konovalova A, Mitchell AM, Silhavy TJ. 2016. A lipoprotein/β-barrel complex monitors lipopolysaccharide integrity transducing information across the outer membrane. eLife 5:e15276. doi:10.7554/eLife.15276 27282389 PMC4942254

[15] Hews CL, Cho T, Rowley G, Raivio TL. 2019. Maintaining integrity under stress: envelope stress response regulation of pathogenesis in Gram-negative bacteria. Front Cell Infect Microbiol 9:313. doi:10.3389/fcimb.2019.00313 31552196 PMC6737893

[16] Dong JM, Iuchi S, Kwan HS, Lu Z, Lin ECC. 1993. The deduced amino-acid sequence of the cloned cpxR gene suggests the protein is the cognate regulator for the membrane sensor, CpxA, in a two-component signal transduction system of Escherichia coli. Gene 136:227–230. doi:10.1016/0378-1119(93)90469-j 8294007

[17] Hung DL, Raivio TL, Jones CH, Silhavy TJ, Hultgren SJ. 2001. Cpx signaling pathway monitors biogenesis and affects assembly and expression of P pili. EMBO J 20:1508–1518. doi:10.1093/emboj/20.7.1508 11285215 PMC145513

[18] Lee YM, DiGiuseppe PA, Silhavy TJ, Hultgren SJ. 2004. P pilus assembly motif necessary for activation of the CpxRA pathway by PapE in Escherichia coli. J Bacteriol 186:4326–4337. doi:10.1128/JB.186.13.4326-4337.2004 15205435 PMC421624

[19] May KL, Lehman KM, Mitchell AM, Grabowicz M. 2019. A stress response monitoring lipoprotein trafficking to the outer membrane. mBio 10:10–1128. doi:10.1128/mBio.00618-19 PMC6538781 31138744

[20] Delhaye A, Laloux G, Collet JF. 2019. The lipoprotein NlpE is a Cpx sensor that serves as a sentinel for protein sorting and folding defects in the Escherichia coli envelope. J Bacteriol 201:10–1128. doi:10.1128/JB.00611-18 PMC6482929 30833359

[21] Xu Y, Zhao Z, Tong W, Ding Y, Liu B, Shi Y, Wang J, Sun S, Liu M, Wang Y, Qi Q, Xian M, Zhao G. 2020. An acid-tolerance response system protecting exponentially growing Escherichia coli. Nat Commun 11:1496. doi:10.1038/s41467-020-15350-5 32198415 PMC7083825

[22] Cho THS, Wang J, Raivio TL. 2023. NlpE is an OmpA-associated outer membrane sensor of the Cpx envelope stress response. J Bacteriol 205:e0040722. doi:10.1128/jb.00407-22 37022159 PMC10127795

[23] Pogliano J, Lynch AS, Belin D, Lin EC, Beckwith J. 1997. Regulation of Escherichia coli cell envelope proteins involved in protein folding and degradation by the Cpx two-component system. Genes Dev 11:1169–1182. doi:10.1101/gad.11.9.1169 9159398

[24] Danese PN, Silhavy TJ. 1997. The sigma(E) and the Cpx signal transduction systems control the synthesis of periplasmic protein-folding enzymes in Escherichia coli. Genes Dev 11:1183–1193. doi:10.1101/gad.11.9.1183 9159399

[25] Yamamoto K, Ishihama A. 2006. Characterization of copper-inducible promoters regulated by CpxA/CpxR in Escherichia coli. Biosci Biotechnol Biochem 70:1688–1695. doi:10.1271/bbb.60024 16861804

[26] Price NL, Raivio TL. 2009. Characterization of the Cpx regulon in Escherichia coli strain MC4100. J Bacteriol 191:1798–1815. doi:10.1128/JB.00798-08 19103922 PMC2648356

[27] Miki T, Ito M, Okada N, Haneda T. 2024. The CpxRA two-component system of adherent and invasive Escherichia coli contributes to epithelial cell invasion and early-stage intestinal fitness in a dysbiotic mouse model mediated by type 1 fimbriae expression. Infect Immun 92:e0013224. doi:10.1128/iai.00132-24 38700334 PMC11237727

[28] Herbert EE, Cowles KN, Goodrich-Blair H. 2007. CpxRA regulates mutualism and pathogenesis in Xenorhabdus nematophila. Appl Environ Microbiol 73:7826–7836. doi:10.1128/AEM.01586-07 17951441 PMC2168154

[29] Leuko S, Raivio TL. 2012. Mutations that impact the enteropathogenic Escherichia coli Cpx envelope stress response attenuate virulence in Galleria mellonella. Infect Immun 80:3077–3085. doi:10.1128/IAI.00081-12 22710873 PMC3418753

[30] Debnath I, Norton JP, Barber AE, Ott EM, Dhakal BK, Kulesus RR, Mulvey MA. 2013. The Cpx stress response system potentiates the fitness and virulence of uropathogenic Escherichia coli. Infect Immun 81:1450–1459. doi:10.1128/IAI.01213-12 23429541 PMC3647988

[31] Dorel C, Lejeune P, Rodrigue A. 2006. The Cpx system of Escherichia coli, a strategic signaling pathway for confronting adverse conditions and for settling biofilm communities? Res Microbiol 157:306–314. doi:10.1016/j.resmic.2005.12.003 16487683

[32] Macritchie DM, Ward JD, Nevesinjac AZ, Raivio TL. 2008. Activation of the Cpx envelope stress response down-regulates expression of several locus of enterocyte effacement-encoded genes in enteropathogenic Escherichia coli. Infect Immun 76:1465–1475. doi:10.1128/IAI.01265-07 18227171 PMC2292881

[33] Dudin O, Geiselmann J, Ogasawara H, Ishihama A, Lacour S. 2014. Repression of flagellar genes in exponential phase by CsgD and CpxR, two crucial modulators of Escherichia coli biofilm formation. J Bacteriol 196:707–715. doi:10.1128/JB.00938-13 24272779 PMC3911157

[34] Rodríguez-Valverde D, León-Montes N, Siqueiros-Cendón T, Rivera-Gutiérrez S, Ares MA, De la Cruz MA. 2023. The CpxRA two-component system represses gene expression of the heat-labile toxin of enterotoxigenic Escherichia coli. J Med Microbiol 72:1682. doi:10.1099/jmm.0.001682 37043376

[35] De la Cruz MA, Ruiz-Tagle A, Ares MA, Pacheco S, Yáñez JA, Cedillo L, Torres J, Girón JA. 2017. The expression of Longus type 4 pilus of enterotoxigenic Escherichia coli is regulated by LngR and LngS and by H-NS, CpxR and CRP global regulators. Environ Microbiol 19:1761–1775. doi:10.1111/1462-2920.13644 27943535

[36] Jacob-Dubuisson F, Pinkner J, Xu Z, Striker R, Padmanhaban A, Hultgren SJ. 1994. PapD chaperone function in pilus biogenesis depends on oxidant and chaperone-like activities of DsbA. Proc Natl Acad Sci USA 91:11552–11556. doi:10.1073/pnas.91.24.11552 7972100 PMC45269

[37] Vogt SL, Nevesinjac AZ, Humphries RM, Donnenberg MS, Armstrong GD, Raivio TL. 2010. The Cpx envelope stress response both facilitates and inhibits elaboration of the enteropathogenic Escherichia coli bundle-forming pilus. Mol Microbiol 76:1095–1110. doi:10.1111/j.1365-2958.2010.07145.x 20444097 PMC2904494

[38] MacRitchie DM, Acosta N, Raivio TL. 2012. DegP is involved in Cpx-mediated posttranscriptional regulation of the type III secretion apparatus in enteropathogenic Escherichia coli. Infect Immun 80:1766–1772. doi:10.1128/IAI.05679-11 22331433 PMC3347454

[39] Tian Z-X, Yi X-X, Cho A, O’Gara F, Wang Y-P. 2016. CpxR activates MexAB-OprM efflux pump expression and enhances antibiotic resistance in both laboratory and clinical nalB-type isolates of Pseudomonas aeruginosa. PLoS Pathog 12:e1005932. doi:10.1371/journal.ppat.1005932 27736975 PMC5063474

[40] Roemhild R, Gokhale CS, Dirksen P, Blake C, Rosenstiel P, Traulsen A, Andersson DI, Schulenburg H. 2018. Cellular hysteresis as a principle to maximize the efficacy of antibiotic therapy. Proc Natl Acad Sci USA 115:9767–9772. doi:10.1073/pnas.1810004115 30209218 PMC6166819

[41] Roy Chowdhury P, Scott M, Worden P, Huntington P, Hudson B, Karagiannis T, Charles IG, Djordjevic SP. 2016. Genomic islands 1 and 2 play key roles in the evolution of extensively drug-resistant ST235 isolates of Pseudomonas aeruginosa. Open Biol 6:150175. doi:10.1098/rsob.150175 26962050 PMC4821235

[42] Singh M, Yau YCW, Wang S, Waters V, Kumar A. 2017. MexXY efflux pump overexpression and aminoglycoside resistance in cystic fibrosis isolates of Pseudomonas aeruginosa from chronic infections. Can J Microbiol 63:929–938. doi:10.1139/cjm-2017-0380 28922614

[43] Tian ZX, Wang YP. 2023. Identification of cpxS mutational resistome in Pseudomonas aeruginosa. Antimicrob Agents Chemother 67:e0092123. doi:10.1128/aac.00921-23 37800959 PMC10648845

[44] Galdino ACM, Vaillancourt M, Celedonio D, Huse K, Doi Y, Lee JS, Jorth P. 2024. Siderophores promote cooperative interspecies and intraspecies cross-protection against antibiotics in vitro. Nat Microbiol 9:631–646. doi:10.1038/s41564-024-01601-4 38409256 PMC11239084

[45] Hirakawa H, Inazumi Y, Masaki T, Hirata T, Yamaguchi A. 2005. Indole induces the expression of multidrug exporter genes in Escherichia coli. Mol Microbiol 55:1113–1126. doi:10.1111/j.1365-2958.2004.04449.x 15686558

[46] Nishino K, Yamasaki S, Hayashi-Nishino M, Yamaguchi A. 2010. Effect of NlpE overproduction on multidrug resistance in Escherichia coli. Antimicrob Agents Chemother 54:2239–2243. doi:10.1128/AAC.01677-09 20211889 PMC2863614

[47] Weatherspoon-Griffin N, Yang D, Kong W, Hua Z, Shi Y. 2014. The CpxR/CpxA two-component regulatory system up-regulates the multidrug resistance cascade to facilitate Escherichia coli resistance to a model antimicrobial peptide. J Biol Chem 289:32571–32582. doi:10.1074/jbc.M114.565762 25294881 PMC4239611

[48] Jones CJ, Grotewold N, Wozniak DJ, Gloag ES. 2022. Pseudomonas aeruginosa initiates a rapid and specific transcriptional response during surface attachment. J Bacteriol 204:e0008622. doi:10.1128/jb.00086-22 35467391 PMC9112911

[49] Harrington NE, Littler JL, Harrison F. 2022. Transcriptome analysis of Pseudomonas aeruginosa biofilm infection in an ex vivo pig model of the cystic fibrosis lung. Appl Environ Microbiol 88:e0178921. doi:10.1128/AEM.01789-21 34878811 PMC8824274

[50] De Wulf P, Akerley BJ, Lin ECC. 2000. Presence of the Cpx system in bacteria. Microbiology (Reading, Engl) 146:247–248. doi:10.1099/00221287-146-2-247 10708362

[51] Mao F, Dam P, Chou J, Olman V, Xu Y. 2009. DOOR: a database for prokaryotic operons. Nucleic Acids Res 37:D459–D463. doi:10.1093/nar/gkn757 18988623 PMC2686520

[52] Winsor GL, Lam DKW, Fleming L, Lo R, Whiteside MD, Yu NY, Hancock REW, Brinkman FSL. 2011. Pseudomonas Genome Database: improved comparative analysis and population genomics capability for Pseudomonas genomes. Nucleic Acids Res 39:D596–D600. doi:10.1093/nar/gkq869 20929876 PMC3013766

[53] Tjaden B. 2020. A computational system for identifying operons based on RNA-seq data. Methods 176:62–70. doi:10.1016/j.ymeth.2019.03.026 30953757 PMC6776731

[54] Mirdita M, Schütze K, Moriwaki Y, Heo L, Ovchinnikov S, Steinegger M. 2022. ColabFold: making protein folding accessible to all. Nat Methods 19:679–682. doi:10.1038/s41592-022-01488-1 35637307 PMC9184281

[55] Bhate MP, Molnar KS, Goulian M, DeGrado WF. 2015. Signal transduction in histidine kinases: insights from new structures. Structure 23:981–994. doi:10.1016/j.str.2015.04.002 25982528 PMC4456306

[56] Orfanoudaki G, Markaki ME, Chatzi KE, Tsamardinos I, Economou A. 2017. MatureP: prediction of secreted proteins with exclusive information from their mature regions. Sci Rep 7:3263. doi:10.1038/s41598-017-03557-4 28607462 PMC5468347

[57] Teufel F, Almagro Armenteros JJ, Johansen AR, Gíslason MH, Pihl SI, Tsirigos KD, Winther O, Brunak S, von Heijne G, Nielsen H. 2022. SignalP 6.0 predicts all five types of signal peptides using protein language models. Nat Biotechnol 40:1023–1025. doi:10.1038/s41587-021-01156-3 34980915 PMC9287161

[58] Yu NY, Wagner JR, Laird MR, Melli G, Rey S, Lo R, Dao P, Sahinalp SC, Ester M, Foster LJ, Brinkman FSL. 2010. PSORTb 3.0: improved protein subcellular localization prediction with refined localization subcategories and predictive capabilities for all prokaryotes. Bioinformatics 26:1608–1615. doi:10.1093/bioinformatics/btq249 20472543 PMC2887053

[59] Danese PN, Silhavy TJ. 1998. CpxP, a stress-combative member of the Cpx regulon. J Bacteriol 180:831–839. doi:10.1128/JB.180.4.831-839.1998 9473036 PMC106961

[60] van Rensburg JJ, Fortney KR, Chen L, Krieger AJ, Lima BP, Wolfe AJ, Katz BP, Zhang Z-Y, Spinola SM. 2015. Development and validation of a high-throughput cell-based screen to identify activators of a bacterial two-component signal transduction system. Antimicrob Agents Chemother 59:3789–3799. doi:10.1128/AAC.00236-15 25870061 PMC4468680

[61] Bury-Moné S, Nomane Y, Reymond N, Barbet R, Jacquet E, Imbeaud S, Jacq A, Bouloc P. 2009. Global analysis of extracytoplasmic stress signaling in Escherichia coli. PLoS Genet 5:e1000651. doi:10.1371/journal.pgen.1000651 19763168 PMC2731931

[62] Taylor DL, Bina XR, Slamti L, Waldor MK, Bina JE. 2014. Reciprocal regulation of resistance-nodulation-division efflux systems and the Cpx two-component system in Vibrio cholerae. Infect Immun 82:2980–2991. doi:10.1128/IAI.00025-14 24799626 PMC4097637

[63] Acosta N, Pukatzki S, Raivio TL. 2015. The Vibrio cholerae Cpx envelope stress response senses and mediates adaptation to low iron. J Bacteriol 197:262–276. doi:10.1128/JB.01957-14 25368298 PMC4272599

[64] Acosta N, Pukatzki S, Raivio TL. 2015. The Cpx system regulates virulence gene expression in Vibrio cholerae. Infect Immun 83:2396–2408. doi:10.1128/IAI.03056-14 25824837 PMC4432731

[65] Raivio TL, Silhavy TJ. 1997. Transduction of envelope stress in Escherichia coli by the Cpx two-component system. J Bacteriol 179:7724–7733. doi:10.1128/jb.179.24.7724-7733.1997 9401031 PMC179735

[66] Raivio TL, Laird MW, Joly JC, Silhavy TJ. 2000. Tethering of CpxP to the inner membrane prevents spheroplast induction of the cpx envelope stress response. Mol Microbiol 37:1186–1197. doi:10.1046/j.1365-2958.2000.02074.x 10972835

[67] Tschauner K, Hörnschemeyer P, Müller VS, Hunke S. 2014. Dynamic interaction between the CpxA sensor kinase and the periplasmic accessory protein CpxP mediates signal recognition in E. coli. PLoS One 9:e107383. doi:10.1371/journal.pone.0107383 25207645 PMC4160245

[68] Quan S, Koldewey P, Tapley T, Kirsch N, Ruane KM, Pfizenmaier J, Shi R, Hofmann S, Foit L, Ren G, Jakob U, Xu Z, Cygler M, Bardwell JCA. 2011. Genetic selection designed to stabilize proteins uncovers a chaperone called Spy. Nat Struct Mol Biol 18:262–269. doi:10.1038/nsmb.2016 21317898 PMC3079333

[69] Keller R, Ariöz C, Hansmeier N, Stenberg-Bruzell F, Burstedt M, Vikström D, Kelly A, Wieslander Å, Daley DO, Hunke S. 2015. The Escherichia coli envelope stress sensor CpxA responds to changes in lipid bilayer properties. Biochemistry 54:3670–3676. doi:10.1021/acs.biochem.5b00242 25993101

[70] Delhaye A, Collet JF, Laloux G. 2016. Fine-tuning of the Cpx envelope stress response is required for cell wall homeostasis in Escherichia coli. mBio 7:e00047-16. doi:10.1128/mBio.00047-16 26908573 PMC4791840

[71] Slamti L, Waldor MK. 2009. Genetic analysis of activation of the Vibrio cholerae Cpx pathway. J Bacteriol 191:5044–5056. doi:10.1128/JB.00406-09 19542291 PMC2725601

[72] Armbruster CR, Lee CK, Parker-Gilham J, de Anda J, Xia A, Zhao K, Murakami K, Tseng BS, Hoffman LR, Jin F, Harwood CS, Wong GC, Parsek MR. 2019. Heterogeneity in surface sensing suggests a division of labor in Pseudomonas aeruginosa populations. eLife 8:e45084. doi:10.7554/eLife.45084 31180327 PMC6615863

[73] Ayoub Moubareck C. 2020. Polymyxins and bacterial membranes: a review of antibacterial activity and mechanisms of resistance. Membranes (Basel) 10:181. doi:10.3390/membranes10080181 32784516 PMC7463838

[74] Larson MH, Gilbert LA, Wang X, Lim WA, Weissman JS, Qi LS. 2013. CRISPR interference (CRISPRi) for sequence-specific control of gene expression. Nat Protoc 8:2180–2196. doi:10.1038/nprot.2013.132 24136345 PMC3922765

[75] Stolle AS, Meader BT, Toska J, Mekalanos JJ. 2021. Endogenous membrane stress induces T6SS activity in Pseudomonas aeruginosa. Proc Natl Acad Sci USA 118:e2018365118. doi:10.1073/pnas.2018365118 33443205 PMC7817224

[76] Ricci DP, Silhavy TJ. 2012. The Bam machine: a molecular cooper. Biochim Biophys Acta 1818:1067–1084. doi:10.1016/j.bbamem.2011.08.020 21893027 PMC3253334

[77] Manat G, Roure S, Auger R, Bouhss A, Barreteau H, Mengin-Lecreulx D, Touzé T. 2014. Deciphering the metabolism of undecaprenyl-phosphate: the bacterial cell-wall unit carrier at the membrane frontier. Microb Drug Resist 20:199–214. doi:10.1089/mdr.2014.0035 24799078 PMC4050452

[78] Melamed J, Kocev A, Torgov V, Veselovsky V, Brockhausen I. 2022. Biosynthesis of the Pseudomonas aeruginosa common polysaccharide antigen by D-rhamnosyltransferases WbpX and WbpY. Glycoconj J 39:393–411. doi:10.1007/s10719-022-10040-4 35166992 PMC8853325

[79] Wang T, Sun W, Fan L, Hua C, Wu N, Fan S, Zhang J, Deng X, Yan J. 2021. An atlas of the binding specificities of transcription factors in Pseudomonas aeruginosa directs prediction of novel regulators in virulence. eLife 10:e61885. doi:10.7554/eLife.61885 33779544 PMC8041468

[80] Guest RL, Wang J, Wong JL, Raivio TL. 2017. A bacterial stress response regulates respiratory protein complexes to control envelope stress adaptation. J Bacteriol 199:10–1128. doi:10.1128/JB.00153-17 PMC5637174 28760851

[81] Lei D, Cao L, Zhong T, He QY, Sun X. 2023. Residue Lys219 of CpxR is critical in the regulation of the antibiotic resistance of Escherichia coli. J Antimicrob Chemother 78:1859–1870. doi:10.1093/jac/dkad171 37288948

[82] Trouillon J, Imbert L, Villard AM, Vernet T, Attrée I, Elsen S. 2021. Determination of the two-component systems regulatory network reveals core and accessory regulations across Pseudomonas aeruginosa lineages. Nucleic Acids Res 49:11476–11490. doi:10.1093/nar/gkab928 34718721 PMC8599809

[83] Love MI, Huber W, Anders S. 2014. Moderated estimation of fold change and dispersion for RNA-seq data with DESeq2. Genome Biol 15:1–21. doi:10.1186/s13059-014-0550-8 PMC4302049 25516281

[84] Shetty D, Abrahante JE, Chekabab SM, Wu X, Korber DR, Vidovic S. 2019. Role of CpxR in biofilm development: expression of key fimbrial, O-antigen and virulence operons of Salmonella enteritidis. Int J Mol Sci 20:5146. doi:10.3390/ijms20205146 31627387 PMC6829429

[85] Otto K, Silhavy TJ. 2002. Surface sensing and adhesion of Escherichia coli controlled by the Cpx-signaling pathway. Proc Natl Acad Sci USA 99:2287–2292. doi:10.1073/pnas.042521699 11830644 PMC122357

[86] Henríquez T, Stein NV, Jung H. 2019. PvdRT-OpmQ and MdtABC-OpmB efflux systems are involved in pyoverdine secretion in Pseudomonas putida KT2440. Environ Microbiol Rep 11:98–106. doi:10.1111/1758-2229.12708 30346656

[87] Poole K, McKay GA. 2003. Iron acquisition and its control in Pseudomonas aeruginosa: many roads lead to Rome. Front Biosci 8:d661–d686. doi:10.2741/1051 12700066

[88] Dieppois G, Ducret V, Caille O, Perron K. 2012. The transcriptional regulator CzcR modulates antibiotic resistance and quorum sensing in Pseudomonas aeruginosa. PLoS One 7:e38148. doi:10.1371/journal.pone.0038148 22666466 PMC3362554

[89] Perron K, Caille O, Rossier C, Van Delden C, Dumas J-L, Köhler T. 2004. CzcR-CzcS, a two-component system involved in heavy metal and carbapenem resistance in Pseudomonas aeruginosa. J Biol Chem 279:8761–8768. doi:10.1074/jbc.M312080200 14679195

[90] Kreamer NNK, Wilks JC, Marlow JJ, Coleman ML, Newman DK. 2012. BqsR/BqsS constitute a two-component system that senses extracellular Fe(II) in Pseudomonas aeruginosa. J Bacteriol 194:1195–1204. doi:10.1128/JB.05634-11 22194456 PMC3294787

[91] Kreamer NN, Costa F, Newman DK. 2015. The ferrous iron-responsive BqsRS two-component system activates genes that promote cationic stress tolerance. mBio 6:e02549. doi:10.1128/mBio.02549-14 25714721 PMC4358008

[92] Michel-Briand Y, Baysse C. 2002. The pyocins of Pseudomonas aeruginosa. Biochimie 84:499–510. doi:10.1016/s0300-9084(02)01422-0 12423794

[93] Chang W, Small DA, Toghrol F, Bentley WE. 2005. Microarray analysis of Pseudomonas aeruginosa reveals induction of pyocin genes in response to hydrogen peroxide. BMC Genomics 6:1–14. doi:10.1186/1471-2164-6-115 16150148 PMC1250226

[94] Cirz RT, O’Neill BM, Hammond JA, Head SR, Romesberg FE. 2006. Defining the Pseudomonas aeruginosa SOS response and its role in the global response to the antibiotic ciprofloxacin. J Bacteriol 188:7101–7110. doi:10.1128/JB.00807-06 17015649 PMC1636241

[95] Bradley JM, Svistunenko DA, Wilson MT, Hemmings AM, Moore GR, Le Brun NE. 2020. Bacterial iron detoxification at the molecular level. J Biol Chem 295:17602–17623. doi:10.1074/jbc.REV120.007746 33454001 PMC7762939

[96] Arai H, Igarashi Y, Kodama T. 1994. Structure and ANR-dependent transcription of the nir genes for denitrification from Pseudomonas aeruginosa. Biosci Biotechnol Biochem 58:1286–1291. doi:10.1271/bbb.58.1286 7765251

[97] Guest RL, Court EA, Waldon JL, Schock KA, Raivio TL. 2019. Impaired efflux of the siderophore enterobactin induces envelope stress in Escherichia coli. Front Microbiol 10:2776. doi:10.3389/fmicb.2019.02776 31866967 PMC6908949

[98] Tsviklist V, Guest RL, Raivio TL. 2021. The Cpx stress response regulates turnover of respiratory chain proteins at the inner membrane of Escherichia coli. Front Microbiol 12:732288. doi:10.3389/fmicb.2021.732288 35154019 PMC8831704

[99] Kimkes TEP, Heinemann M. 2018. Reassessing the role of the Escherichia coli CpxAR system in sensing surface contact. PLoS One 13:e0207181. doi:10.1371/journal.pone.0207181 30412611 PMC6226299

[100] Shimizu T, Ichimura K, Noda M. 2016. The surface sensor NlpE of enterohemorrhagic Escherichia coli contributes to regulation of the type III secretion system and flagella by the Cpx response to adhesion. Infect Immun 84:537–549. doi:10.1128/IAI.00881-15 26644384 PMC4730559

[101] Cho THS, Murray C, Malpica R, Margain-Quevedo R, Thede GL, Lu J, Edwards RA, Glover JNM, Raivio TL. 2024. The sensor of the bacterial histidine kinase CpxA is a novel dimer of extracytoplasmic Per-ARNT-Sim domains. J Biol Chem 300:107265. doi:10.1016/j.jbc.2024.107265 38582452 PMC11078701

[102] Valentini M, Filloux A. 2016. Biofilms and cyclic di-GMP (c-di-GMP) signaling: lessons from Pseudomonas aeruginosa and other bacteria. J Biol Chem 291:12547–12555. doi:10.1074/jbc.R115.711507 27129226 PMC4933438

[103] Dartigalongue C, Missiakas D, Raina S. 2001. Characterization of the Escherichia coliς^E^ regulon. J Biol Chem 276:20866–20875. doi:10.1074/jbc.M100464200 11274153

[104] Wood LF, Ohman DE. 2009. Use of cell wall stress to characterize σ^22^ (AlgT/U) activation by regulated proteolysis and its regulon in Pseudomonas aeruginosa. Mol Microbiol 72:183–201. doi:10.1111/j.1365-2958.2009.06635.x 19226327

[105] Wood LF, Ohman DE. 2012. Identification of genes in the σ^22^ regulon of Pseudomonas aeruginosa required for cell envelope homeostasis in either the planktonic or the sessile mode of growth. mBio 3:e00094-12. doi:10.1128/mBio.00094-12 22589289 PMC3372973

[106] Rhodius VA, Suh WC, Nonaka G, West J, Gross CA. 2006. Conserved and variable functions of the σ^E^ stress response in related genomes. PLoS Biol 4:e2. doi:10.1371/journal.pbio.0040002 16336047 PMC1312014

[107] Rowley G, Spector M, Kormanec J, Roberts M. 2006. Pushing the envelope: extracytoplasmic stress responses in bacterial pathogens. Nat Rev Microbiol 4:383–394. doi:10.1038/nrmicro1394 16715050

[108] Ramsey DM, Wozniak DJ. 2005. Understanding the control of Pseudomonas aeruginosa alginate synthesis and the prospects for management of chronic infections in cystic fibrosis. Mol Microbiol 56:309–322. doi:10.1111/j.1365-2958.2005.04552.x 15813726

[109] Sultan M, Arya R, Kim KK. 2021. Roles of two-component systems in Pseudomonas aeruginosa virulence. Int J Mol Sci 22:12152. doi:10.3390/ijms222212152 34830033 PMC8623646

[110] Poole K, Hay T, Gilmour C, Fruci M. 2019. The aminoglycoside resistance-promoting AmgRS envelope stress-responsive two-component system in Pseudomonas aeruginosa is zinc-activated and protects cells from zinc-promoted membrane damage. Microbiology (Reading) 165:563–571. doi:10.1099/mic.0.000787 30835196

[111] Lau CH-F, Krahn T, Gilmour C, Mullen E, Poole K. 2015. AmgRS-mediated envelope stress-inducible expression of the mexXY multidrug efflux operon of Pseudomonas aeruginosa. Microbiologyopen 4:121–135. doi:10.1002/mbo3.226 25450797 PMC4335980

[112] Lee S, Hinz A, Bauerle E, Angermeyer A, Juhaszova K, Kaneko Y, Singh PK, Manoil C. 2009. Targeting a bacterial stress response to enhance antibiotic action. Proc Natl Acad Sci USA 106:14570–14575. doi:10.1073/pnas.0903619106 19706543 PMC2732827

[113] Yu L, Cao Q, Chen W, Yang N, Yang CG, Ji Q, Wu M, Bae T, Lan L. 2022. A novel copper-sensing two-component system for inducing Dsb gene expression in bacteria. Sci Bull Sci Found Philipp 67:198–212. doi:10.1016/j.scib.2021.03.003 36546013

[114] Wei X, Gao J, Xu C, Pan X, Jin Y, Bai F, Cheng Z, Lamont IL, Pletzer D, Wu W. 2023. Murepavadin induces envelope stress response and enhances the killing efficacies of β-lactam antibiotics by impairing the outer membrane integrity of Pseudomonas aeruginosa. Microbiol Spectr 11:e0125723. doi:10.1128/spectrum.01257-23 37668398 PMC10581190

[115] Gellatly SL, Needham B, Madera L, Trent MS, Hancock REW. 2012. The Pseudomonas aeruginosa PhoP-PhoQ two-component regulatory system is induced upon interaction with epithelial cells and controls cytotoxicity and inflammation. Infect Immun 80:3122–3131. doi:10.1128/IAI.00382-12 22710876 PMC3418734

[116] Monteagudo-Cascales E, García-Mauriño SM, Santero E, Canosa I. 2019. Unraveling the role of the CbrA histidine kinase in the signal transduction of the CbrAB two-component system in Pseudomonas putida. Sci Rep 9:9110. doi:10.1038/s41598-019-45554-9 31235731 PMC6591292

[117] Gessmann D, Chung YH, Danoff EJ, Plummer AM, Sandlin CW, Zaccai NR, Fleming KG. 2014. Outer membrane β-barrel protein folding is physically controlled by periplasmic lipid head groups and BamA. Proc Natl Acad Sci USA 111:5878–5883. doi:10.1073/pnas.1322473111 24715731 PMC4000854

[118] Storek KM, Sun D, Rutherford ST. 2024. Inhibitors targeting BamA in gram-negative bacteria. Biochim Biophys Acta Mol Cell Res 1871:119609. doi:10.1016/j.bbamcr.2023.119609 37852326

[119] Gerken H, Leiser OP, Bennion D, Misra R. 2010. Involvement and necessity of the Cpx regulon in the event of aberrant β-barrel outer membrane protein assembly. Mol Microbiol 75:1033–1046. doi:10.1111/j.1365-2958.2009.07042.x 20487295 PMC2927739

[120] Grabowicz M, Koren D, Silhavy TJ. 2016. The CpxQ sRNA negatively regulates Skp to prevent mistargeting of β-barrel outer membrane proteins into the cytoplasmic membrane. mBio 7:e00312-16. doi:10.1128/mBio.00312-16 27048800 PMC4817254

[121] Ma Q, Wood TK. 2009. OmpA influences Escherichia coli biofilm formation by repressing cellulose production through the CpxRA two-component system. Environ Microbiol 11:2735–2746. doi:10.1111/j.1462-2920.2009.02000.x 19601955

[122] Lorenz C, Dougherty TJ, Lory S. 2020. Transcriptional responses of Pseudomonas aeruginosa to inhibition of lipoprotein transport by a small molecule inhibitor. J Bacteriol 202:e00452-20. doi:10.1128/JB.00452-20 32989085 PMC7685553

[123] Nickerson NN, Jao CC, Xu Y, Quinn J, Skippington E, Alexander MK, Miu A, Skelton N, Hankins JV, Lopez MS, Koth CM, Rutherford S, Nishiyama M. 2018. A novel inhibitor of the LolCDE ABC transporter essential for lipoprotein trafficking in Gram-negative bacteria. Antimicrob Agents Chemother 62:10–1128. doi:10.1128/AAC.02151-17 PMC5913989 29339384

[124] Lorenz C, Dougherty TJ, Lory S. 2019. Correct sorting of lipoproteins into the inner and outer membranes of Pseudomonas aeruginosa by the Escherichia coli LolCDE transport system. mBio 10:e00194-19. doi:10.1128/mBio.00194-19 30992347 PMC6469965

[125] Freschi L, Jeukens J, Kukavica-Ibrulj I, Boyle B, Dupont M-J, Laroche J, Larose S, Maaroufi H, Fothergill JL, Moore M, et al.. 2015. Clinical utilization of genomics data produced by the international Pseudomonas aeruginosa consortium. Front Microbiol 6:1036. doi:10.3389/fmicb.2015.01036 26483767 PMC4586430

[126] Kasetty S, Katharios-Lanwermeyer S, O’Toole GA, Nadell CD. 2021. Differential surface competition and biofilm invasion strategies of Pseudomonas aeruginosa PA14 and PAO1. J Bacteriol 203:e0026521. doi:10.1128/JB.00265-21 34516283 PMC8544417

[127] Lee CK, Vachier J, de Anda J, Zhao K, Baker AE, Bennett RR, Armbruster CR, Lewis KA, Tarnopol RL, Lomba CJ, Hogan DA, Parsek MR, O’Toole GA, Golestanian R, Wong GCL. 2020. Social cooperativity of bacteria during reversible surface attachment in young biofilms: a quantitative comparison of Pseudomonas aeruginosa PA14 and PAO1. mBio 11:e02644-19. doi:10.1128/mBio.02644-19 32098815 PMC7042694

[128] Caiazza NC, O’Toole GA. 2004. SadB is required for the transition from reversible to irreversible attachment during biofilm formation by Pseudomonas aeruginosa PA14. J Bacteriol 186:4476–4485. doi:10.1128/JB.186.14.4476-4485.2004 15231779 PMC438627

[129] Banin E, Vasil ML, Greenberg EP. 2005. Iron and Pseudomonas aeruginosa biofilm formation. Proc Natl Acad Sci USA 102:11076–11081. doi:10.1073/pnas.0504266102 16043697 PMC1182440

[130] Zhang Y, Pan X, Wang L, Chen L. 2021. Iron metabolism in Pseudomonas aeruginosa biofilm and the involved iron-targeted anti-biofilm strategies. J Drug Target 29:249–258. doi:10.1080/1061186X.2020.1824235 32969723

[131] Goodman AE, Marshall KC. 1995. Genetic responses of bacteria at surfaces, p 80–98. In Costerton JW, Lappin-Scott HM (ed), Microbial biofilms. Cambridge University Press, Cambridge.

[132] Hmelo LR, Borlee BR, Almblad H, Love ME, Randall TE, Tseng BS, Lin C, Irie Y, Storek KM, Yang JJ, Siehnel RJ, Howell PL, Singh PK, Tolker-Nielsen T, Parsek MR, Schweizer HP, Harrison JJ. 2015. Precision-engineering the Pseudomonas aeruginosa genome with two-step allelic exchange. Nat Protoc 10:1820–1841. doi:10.1038/nprot.2015.115 26492139 PMC4862005

[133] Huang W, Wilks A. 2017. A rapid seamless method for gene knockout in Pseudomonas aeruginosa. BMC Microbiol 17:199. doi:10.1186/s12866-017-1112-5 28927382 PMC5606073

[134] Newman JR, Fuqua C. 1999. Broad-host-range expression vectors that carry the l-arabinose-inducible Escherichia coli araBAD promoter and the araC regulator. Gene 227:197–203. doi:10.1016/S0378-1119(98)00601-5 10023058

[135] Choi KH, Kumar A, Schweizer HP. 2006. A 10-min method for preparation of highly electrocompetent Pseudomonas aeruginosa cells: application for DNA fragment transfer between chromosomes and plasmid transformation. J Microbiol Methods 64:391–397. doi:10.1016/j.mimet.2005.06.001 15987659

[136] Choi KH, Schweizer HP. 2006. Mini-Tn7 insertion in bacteria with single attTn7 sites: example Pseudomonas aeruginosa. Nat Protoc 1:153–161. doi:10.1038/nprot.2006.24 17406227

[137] Dymecki SM. 1996. A modular set of Flp, FRT and lacZ fusion vectors for manipulating genes by site-specific recombination. Gene 171:197–201. doi:10.1016/0378-1119(96)00035-2 8666272

[138] Griffith KL, Wolf RE. 2002. Measuring β-galactosidase activity in bacteria: cell growth, permeabilization, and enzyme assays in 96-well arrays. Biochem Biophys Res Commun 290:397–402. doi:10.1006/bbrc.2001.6152 11779182

[139] Kovach ME, Phillips RW, Elzer PH, Roop RM, Peterson KM. 1994. pBBR1MCS: a broad-host-range cloning vector. BioTechniques 16:800–802. 8068328

[140] Campbell BC, Nabel EM, Murdock MH, Lao-Peregrin C, Tsoulfas P, Blackmore MG, Lee FS, Liston C, Morishita H, Petsko GA. 2020. mGreenLantern: a bright monomeric fluorescent protein with rapid expression and cell filling properties for neuronal imaging. Proc Natl Acad Sci USA 117:30710–30721. doi:10.1073/pnas.2000942117 33208539 PMC7720163

[141] Andersen JB, Sternberg C, Poulsen LK, Bjorn SP, Givskov M, Molin S. 1998. New unstable variants of green fluorescent protein for studies of transient gene expression in bacteria. Appl Environ Microbiol 64:2240–2246. doi:10.1128/AEM.64.6.2240-2246.1998 9603842 PMC106306

[142] Bindels DS, Haarbosch L, van Weeren L, Postma M, Wiese KE, Mastop M, Aumonier S, Gotthard G, Royant A, Hink MA, Gadella TJ. 2017. mScarlet: a bright monomeric red fluorescent protein for cellular imaging. Nat Methods 14:53–56. doi:10.1038/nmeth.4074 27869816

[143] Ducret A, Quardokus EM, Brun YV. 2016. MicrobeJ, a tool for high throughput bacterial cell detection and quantitative analysis. Nat Microbiol 1:16077. doi:10.1038/nmicrobiol.2016.77 27572972 PMC5010025

[144] Friedman L, Kolter R. 2004. Genes involved in matrix formation in Pseudomonas aeruginosa PA14 biofilms. Mol Microbiol 51:675–690. doi:10.1046/j.1365-2958.2003.03877.x 14731271

[145] Jacobs HM, O’Neal L, Lopatto E, Wozniak DJ, Bjarnsholt T, Parsek MR. 2022. Mucoid Pseudomonas aeruginosa can produce calcium-gelled biofilms independent of the matrix components Psl and CdrA. J Bacteriol 204:e0056821. doi:10.1128/jb.00568-21 35416688 PMC9112934

[146] Bragonzi A, Wiehlmann L, Klockgether J, Cramer N, Worlitzsch D, Döring G, Tümmler B. 2006. Sequence diversity of the mucABD locus in Pseudomonas aeruginosa isolates from patients with cystic fibrosis. Microbiology (Reading) 152:3261–3269. doi:10.1099/mic.0.29175-0 17074897

[147] Zhang Y, Liu T, Meyer CA, Eeckhoute J, Johnson DS, Bernstein BE, Nusbaum C, Myers RM, Brown M, Li W, Liu XS. 2008. Model-based analysis of ChIP-Seq (MACS). Genome Biol 9:1–9. doi:10.1186/gb-2008-9-9-r137 PMC2592715 18798982

[148] Quinlan AR, Hall IM. 2010. BEDTools: a flexible suite of utilities for comparing genomic features. Bioinformatics 26:841–842. doi:10.1093/bioinformatics/btq033 20110278 PMC2832824

[149] Bolger AM, Lohse M, Usadel B. 2014. Trimmomatic: a flexible trimmer for Illumina sequence data. Bioinformatics 30:2114–2120. doi:10.1093/bioinformatics/btu170 24695404 PMC4103590

[150] Stover CK, Pham XQ, Erwin AL, Mizoguchi SD, Warrener P, Hickey MJ, Brinkman FSL, Hufnagle WO, Kowalik DJ, Lagrou M, et al.. 2000. Complete genome sequence of Pseudomonas aeruginosa PAO1, an opportunistic pathogen. Nature 406:959–964. doi:10.1038/35023079 10984043

[151] Dobin A, Davis CA, Schlesinger F, Drenkow J, Zaleski C, Jha S, Batut P, Chaisson M, Gingeras TR. 2013. STAR: ultrafast universal RNA-seq aligner. Bioinformatics 29:15–21. doi:10.1093/bioinformatics/bts635 23104886 PMC3530905

[152] Winsor GL, Griffiths EJ, Lo R, Dhillon BK, Shay JA, Brinkman FSL. 2016. Enhanced annotations and features for comparing thousands of Pseudomonas genomes in the Pseudomonas genome database. Nucleic Acids Res 44:D646–D653. doi:10.1093/nar/gkv1227 26578582 PMC4702867

[153] Livak KJ, Schmittgen TD. 2001. Analysis of relative gene expression data using real-time quantitative PCR and the 2^−ΔΔCT^ method. Methods 25:402–408. doi:10.1006/meth.2001.1262 11846609

