# Supplementary material for: The Pseudomonas aeruginosa Cpx system provides a cyclic-di-GMP independent link between cell envelope stress and surface sensing
Source: mBio. 2025 Dec 16;17(1):e02726-25. doi: 10.1128/mbio.02726-25 (PMC12802237; doi:10.1128/mbio.02726-25)
Supplement: Supplemental Figures — Fig. S1 to S13. [file mbio.02726-25-s0001.docx]

**FIGURE S1**


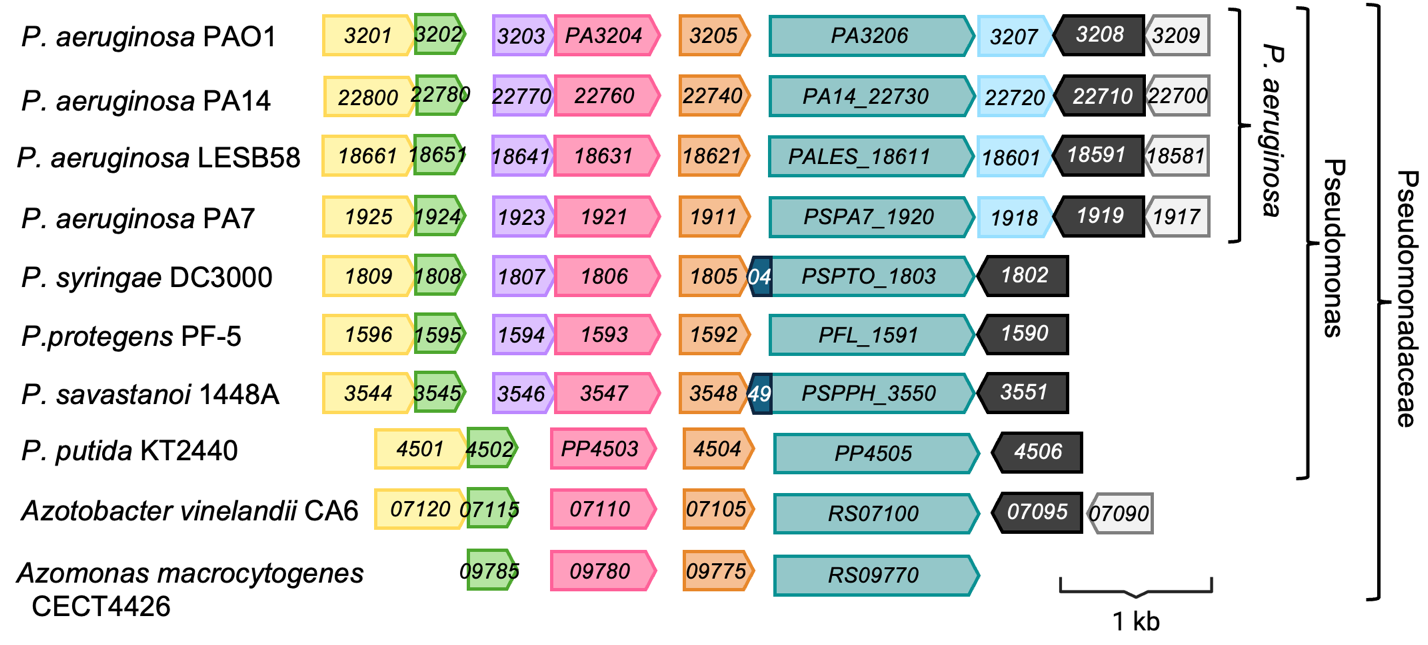


**Fig S1. Incorporation of novel genetic elements in the *P. aeruginosa* *cpx* locus.** In *P. aeruginosa*, hypothetical protein-coding genes form putative operons with two-component system genes *cpxS* and *cpxR*, which are absent from previously characterized Cpx systems in other bacteria. Orthologs of *PA3207* (PAO1), linked to *cpxS*, are conserved throughout the *P. aeruguinosa* species group, while orthologs of *PA3203*, linked to *cpxR*, are generally present throughout the genus Pseudomonas. Bacteria from different genera within Pseudomonadaceae exhibit rearrangement of the Cpx locus relative to the *E. coli* K12 MG1655 model system. Given the presence of well-conserved functional domains across unrelated two-component systems, homologs were identified based on blastp search of PAO1 *cpxP* (*PA3205*) against BioCyc sequence databases, using default settings including the BLOSUM62 scoring matrix (1). Gene cluster schematics are shown to approximate scale with arrows indicating protein coding sequence direction, orthologs grouped by color. Scale bar shows 1 kb.

**FIGURE S2**

**
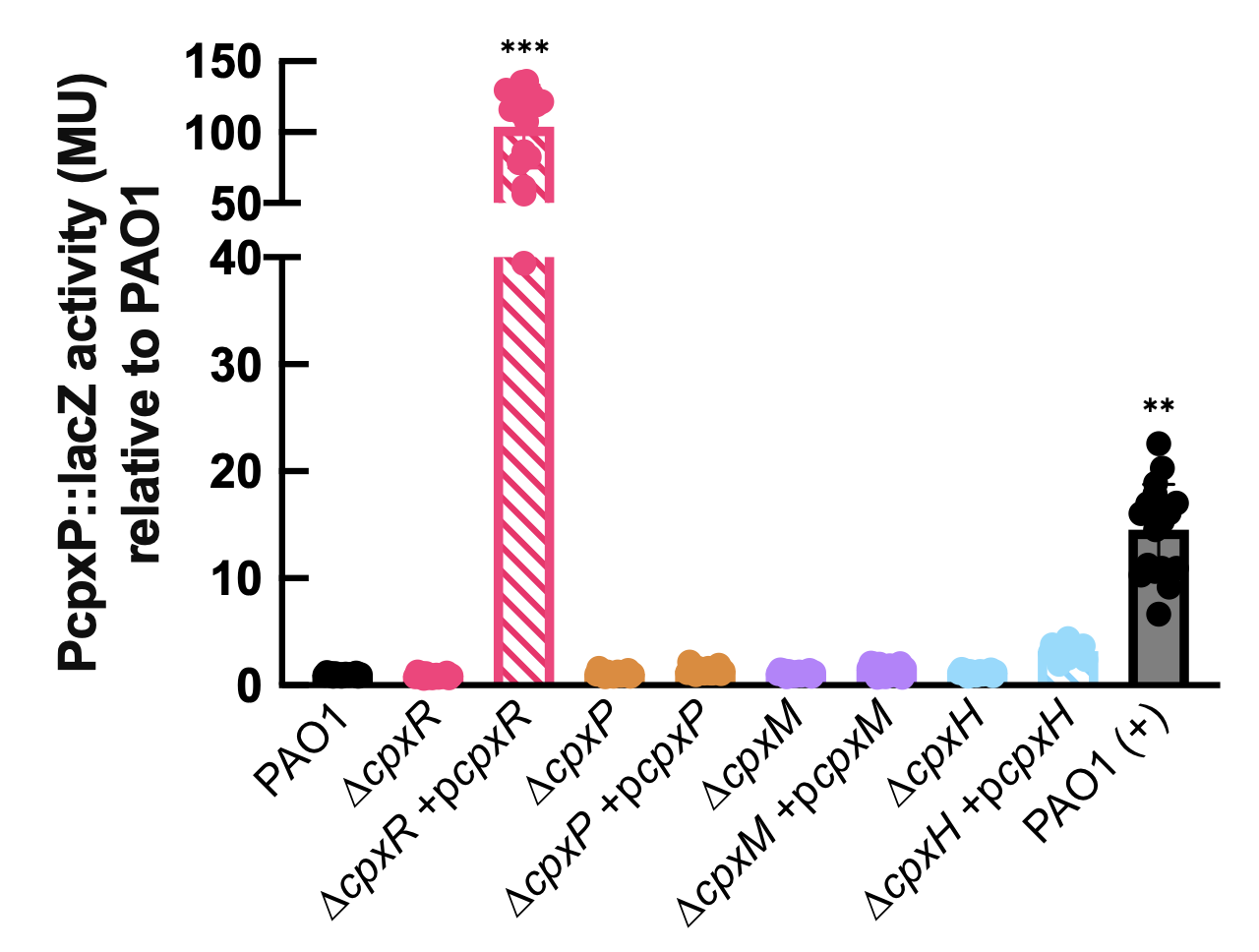
**

**Fig S2. Cpx reporter activity is low during unstressed PAO1 growth.** Extended data from **Fig. 2**. Activity of a Tn*7*::P*_cpxP_*::*lacZ* integrated reporter was measured from cell lysates of PAO1 and derivative *cpx* mutant strains grown 3 h in LB with 100 µg/mL gentamicin. Bacteria carried either an unmodified pJN105 plasmid or pJN105 expressing complementing gene alleles under an araBAD arabinose-inducing expression cassette. 0.1% arabinose was added to media to induce complementing gene expression. Miller Units (MU) were calculated by β-galactosidase assay and normalized to PAO1 levels. For comparison, reporter activity from PAO1 grown in the presence of 2% ethanol (+) is shown. Asterisks indicate statistical significance based on one-way ANOVA with multiple comparisons against PAO1 (unstressed); ** p<0.01; *** p<0.001. Data are pooled from three independent experiments, n=18.

**FIGURE S3**


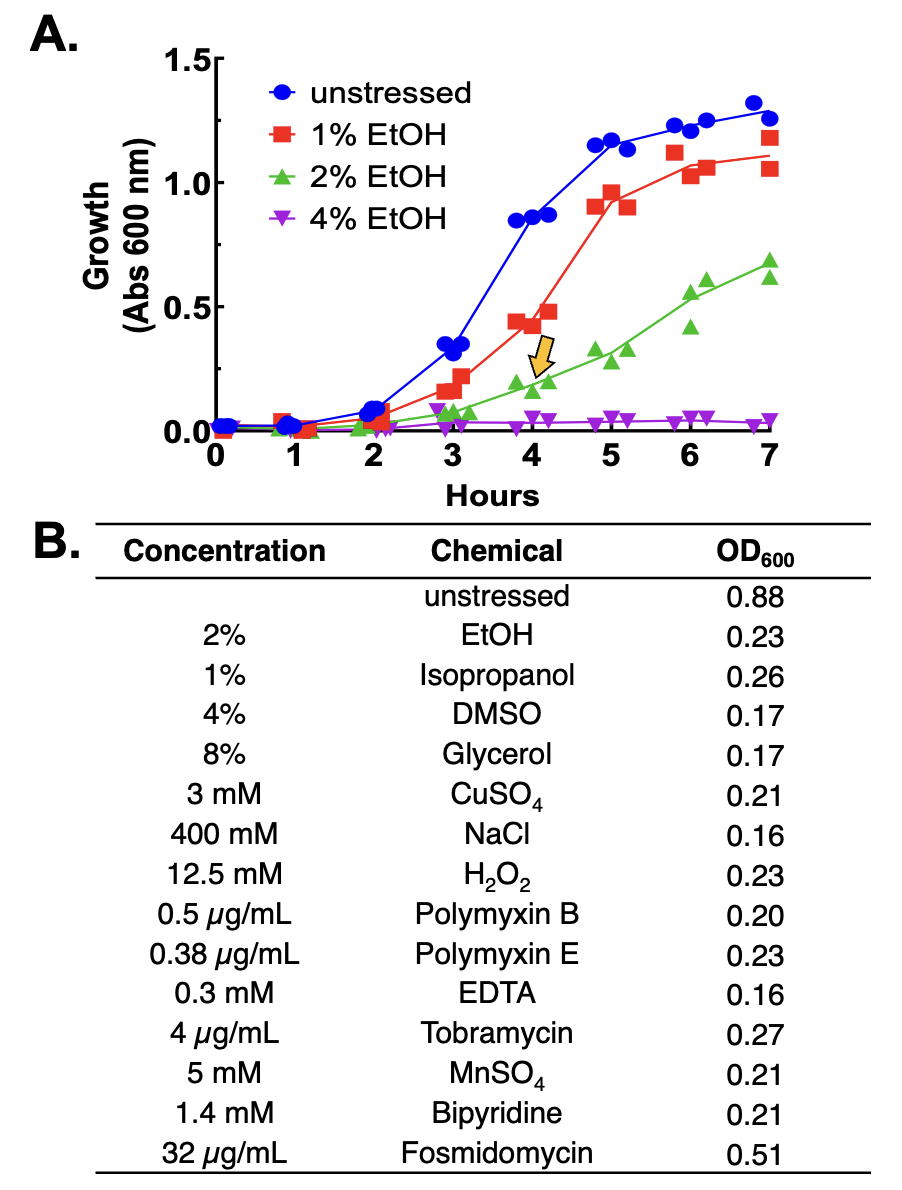


**Fig S3. Selection of chemical stressor concentrations for screening Cpx inducers.** For the chemical stressor screen using single cell fluorescence microscopy, chemical concentrations were chosen that caused a similar growth defect after 4 h. (**A**) Decreasing growth (Abs_600_) of PAO1 expressing the bicolor fluorescent reporter pBBR1 plasmid containing transcriptional fusions of P*_cpxP_*::mGreenLantern/P*_rpoD_*::mScarlet was observed with increasing concentrations of ethanol (EtOH). For all chemicals used in the screen, the concentrations chosen to be used for single cell microscopy caused growth defects similar to 2% EtOH at 4 h (indicated by yellow arrow), except for fosmidomycin ,which was harvested at 4 h at a higher Abs_600_ than the rest of the conditions. (**B**) After 4 h of growth in media containing various chemicals, Abs_600_ was measured by spectrophotometer prior to cell fixation. For each chemical used, the average Abs_600_ value between all replicates is presented.

**FIGURE S4**
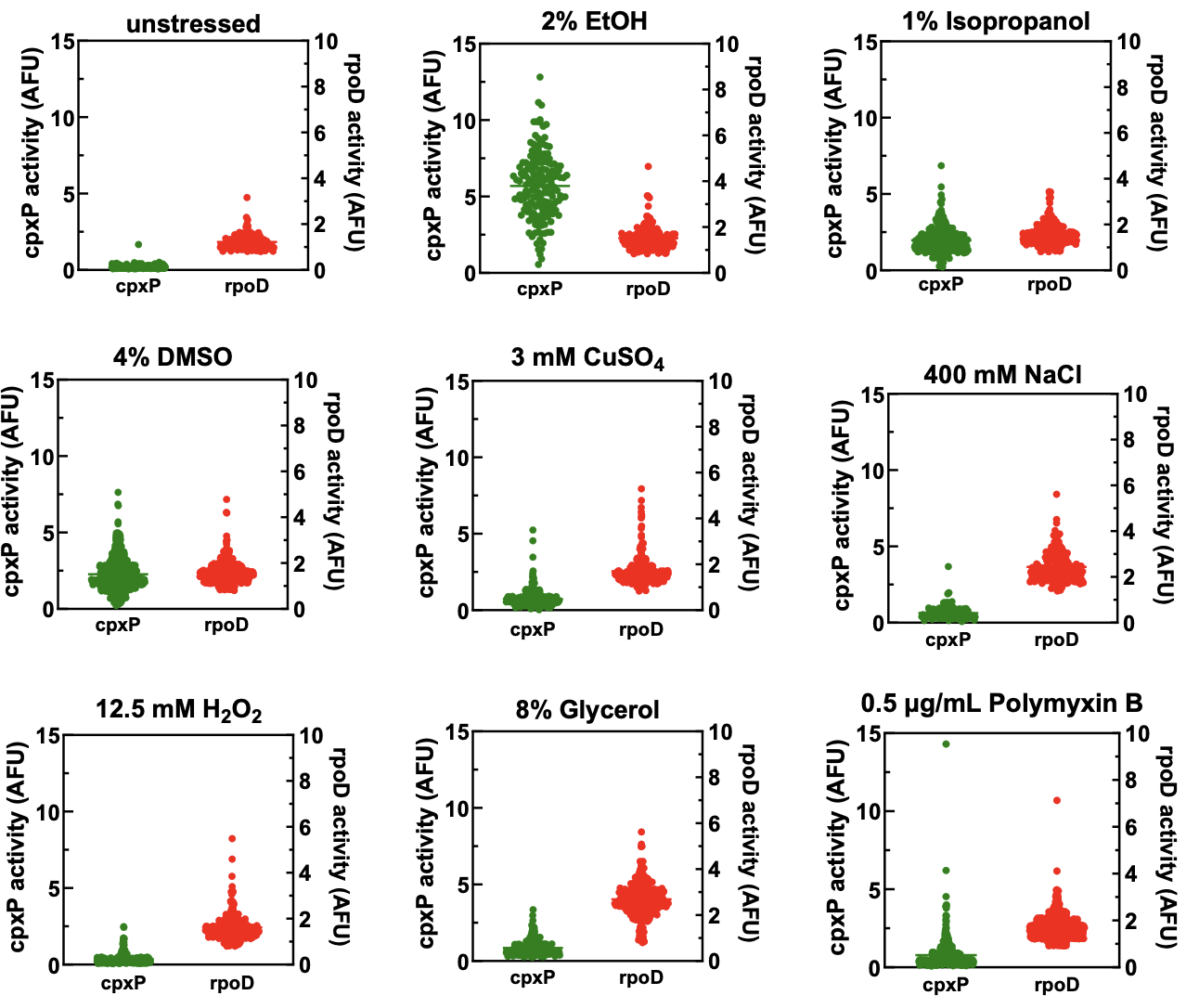

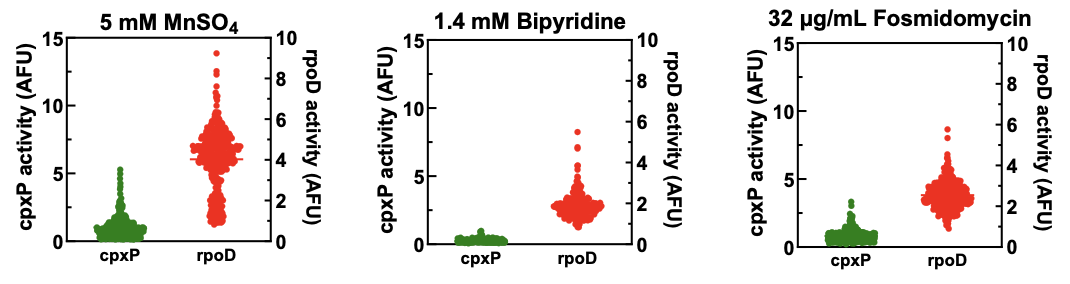


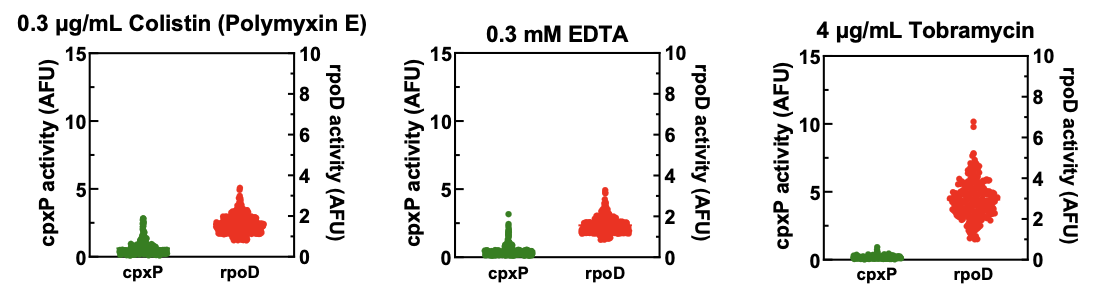


**Fig S4. Raw fluorescence values for PAO1 cells expressing P*_cpxP_*::mGreenLantern/ P*_rpoD_*::mScarlet-I reporters under cell envelope stress**. PAO1 with a bicolor P*_cpxP_*::mGreenLantern/P*_rpoD_*::mScarlet-I reporter plasmid was grown in the presence of the indicated chemical stressors for 4 h, after which cells (>100) were imaged by microscopy. Average background-subtracted fluorescence intensity is shown for each cell as quantified by MicrobeJ (2). Representative samples for each chemical condition are shown. Graphed are single-cell fluorescence intensity, with background florescence subtracted, with green dots representing P*_cpxP_*::mGreenLantern signal and red dots representing P*_rpoD_*::mScarlet-I signal.

**FIGURE S5**


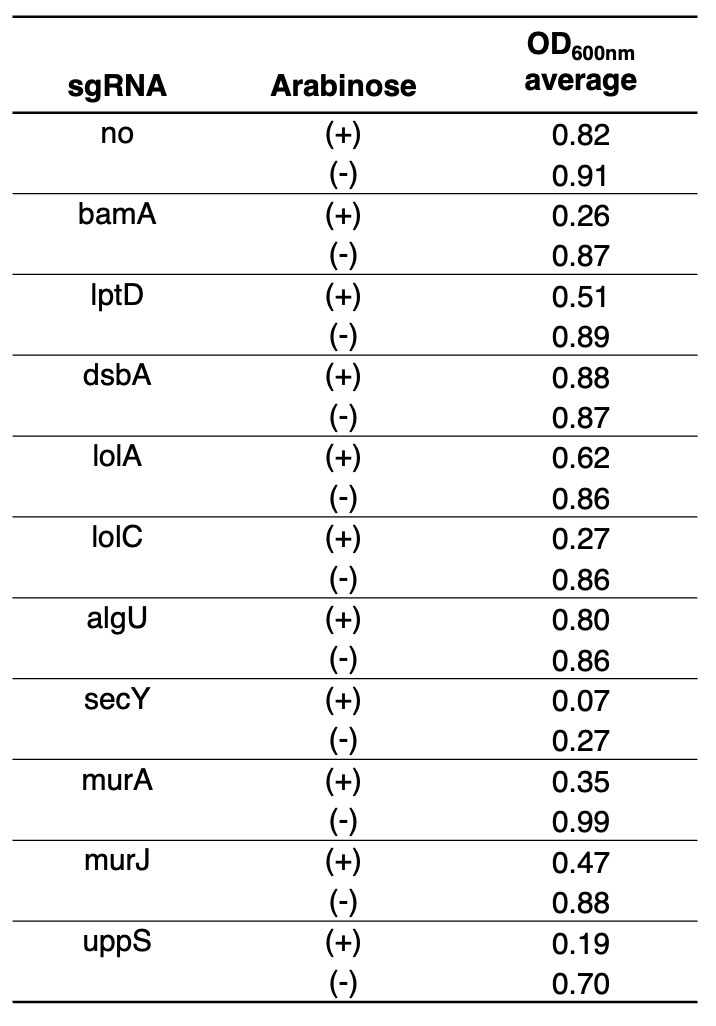


**Fig S5. CRISPRi induction impacts PAO1 growth.** Average Abs_600_ values for each sgRNA. PAO1 strains with CRISPRi cassette and bicolor fluorescence reporter (**Fig. 4**) were grown for 4 hours with (+) and without (-) 0.2% arabinose to induce sp*dcas9* expression. Before harvesting and fixing for single cell microscopy, the absorbance 600 nm was recorded. The average values for all replicates for each sgRNA are presented.

**FIGURE S6**


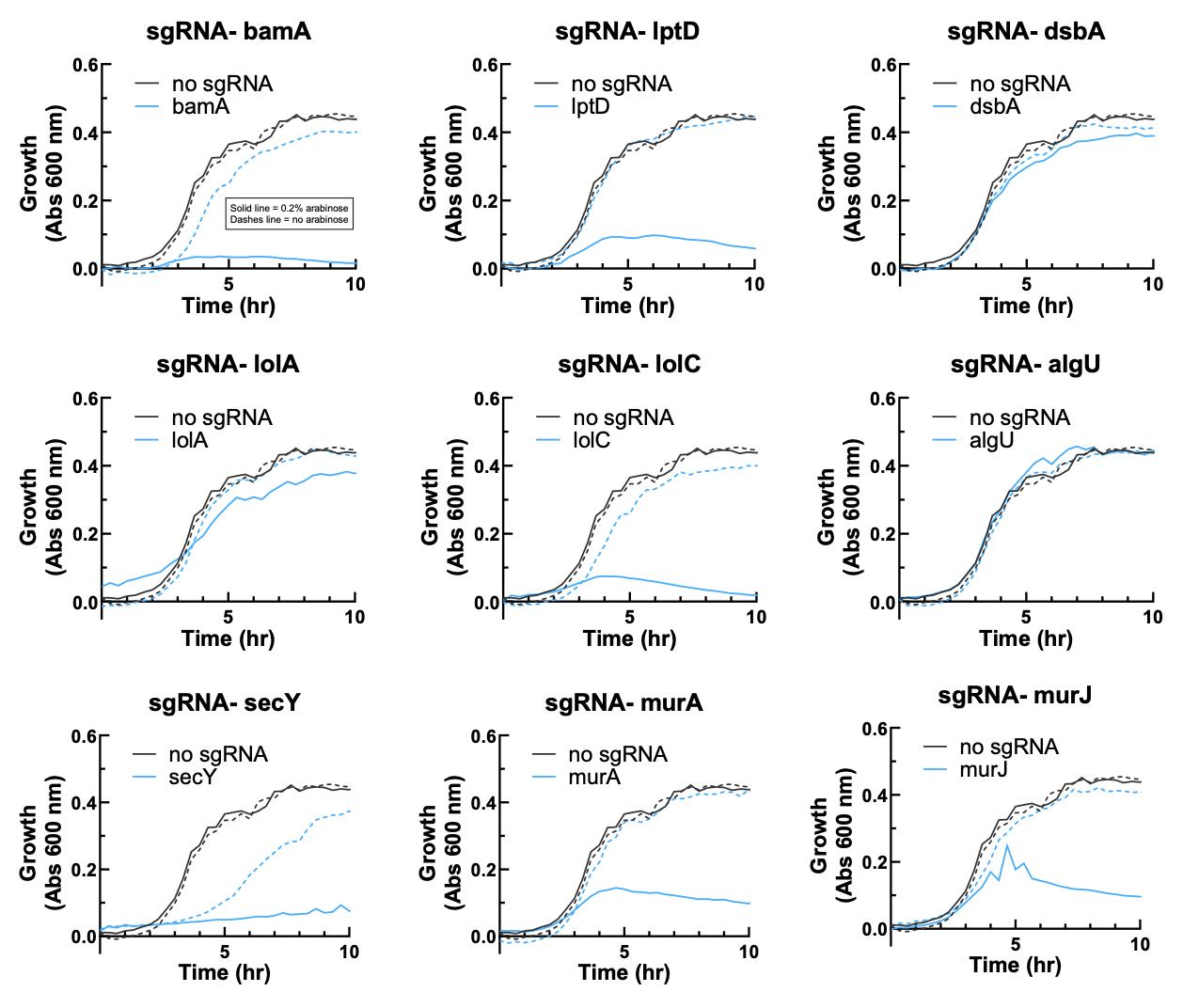


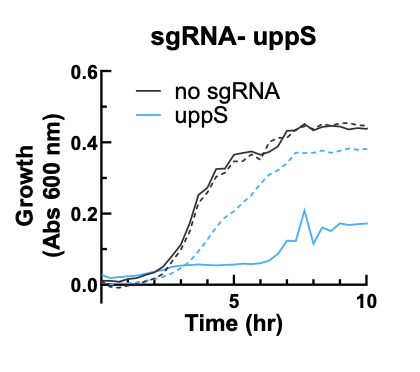


**Fig S6. CRISPRi depletion of essential gene expression attenuates PAO1 growth.** PAO1 Tn*7::araBAD::sp*dcas9 strains expressed a bicolor reporter plasmid containing variable sgRNAs under constitutive promoter control. Bacteria diluted from overnight cultures were grown 10 h in a 96-well microplate in LB (dotted lines) or LB with 0.2% arabinose (solid lines) to induce *sp*dcas9 expression. Absorbance at 600 nm was measured every 20 mins by CLAIROstar PLUS microplate reader. Graphed are mean values from two biological and three technical replicates.

**FIGURE S7**


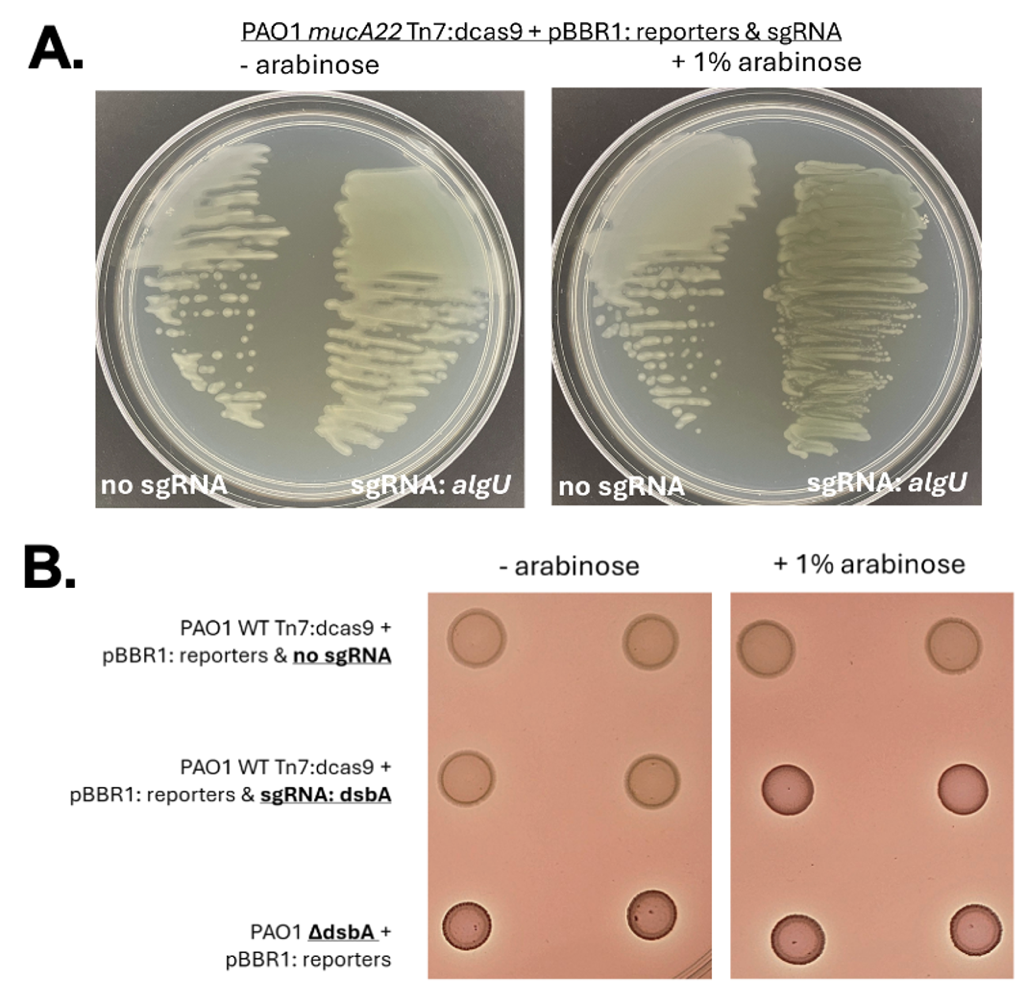


**Fig S7. Phenotypic validation of CRISPRi transcriptional knockdown for nonessential genes in PAO1. (A)** The *mucA22* mutation, conferring a mucoid phenotype, was introduced in PAO1 containing a Tn*7::araBAD::sp*dcas9 cassette. Bacteria expressing a bicolor reporter plasmid containing the *algU* sgRNA (right) or no sgRNA (left) were spotted onto 1.5% Pseudomonas Isolation Agar plates with 100 µg/mL gentamicin, with or without 1% arabinose to induce *sp*dcas9 expression. Loss of the mucoid phenotype is visible in *algU* sgRNA expressing cells under CRISPRi induction (far right). **(B)** Cells expressing the either the *dsbA* sgRNA or no sgRNA, and a PAO1 *ΔdsbA* mutant containing the bicolor fluorescent reporter (no sgRNA), were spotted onto VBMM 1.5% agar plates with 80 µg/mL Congo Red, 30 µg/mL Brilliant Blue, and 100 µg/mL gentamicin. 1% arabinose was added to induce *sp*dcas9 expression. Loss of *dsbA* expression visibly enhances colony staining in the presence of Congo Red and Brilliant Blue, a phenotype observed in both PAO1 *ΔdsbA* and cells expressing the *dsbA* sgRNA under CRISPRi inducing conditions.

**FIGURE S8**


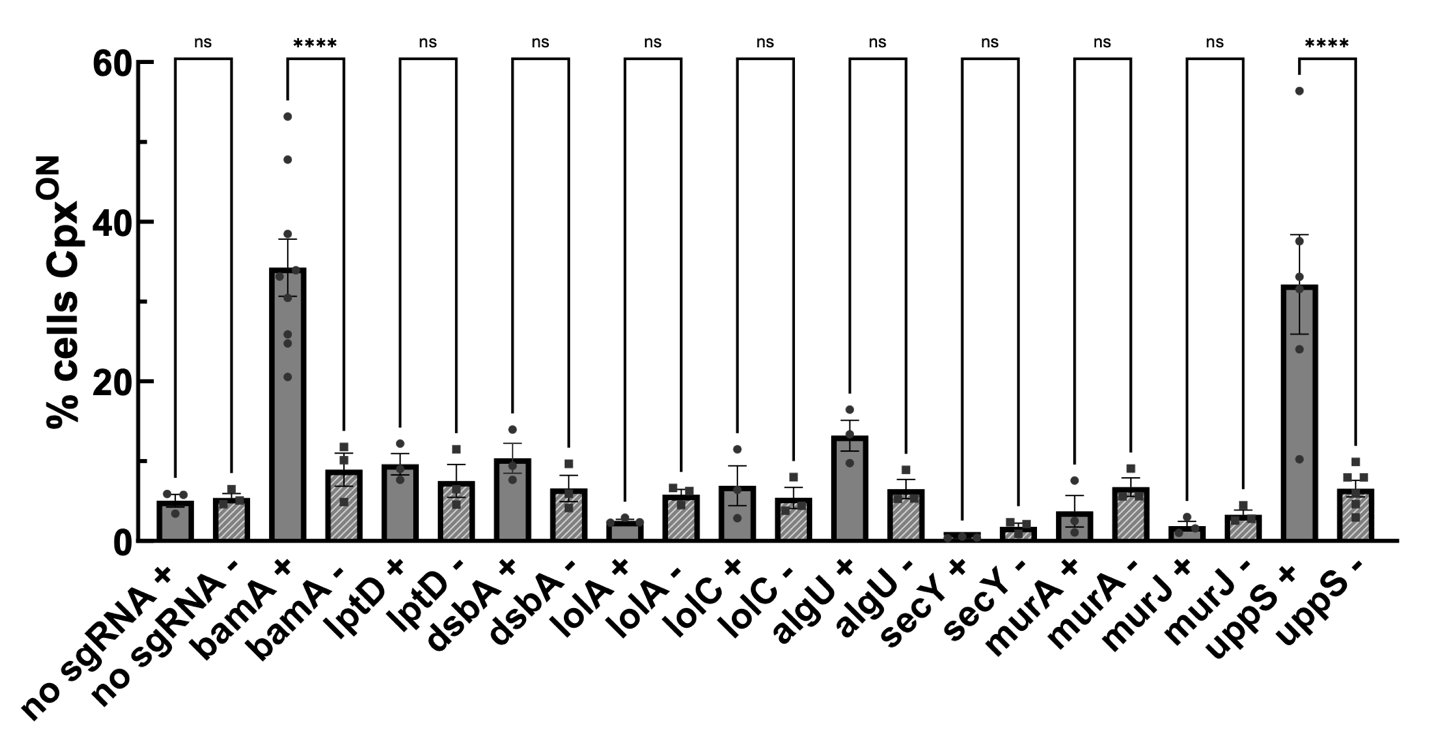


**Fig S8. CRISPRi knockdown of key envelope homeostasis regulatory genes does not impact Cpx signaling when the** sp**dcas9 machinery is not induced.** Extended data from **Fig. 4**. PAO1 T*n*7:: *sp*dcas9 strains expressed a P*_cpxP_*::mGreenLantern/P*_rpoD_*::mScarlet fluorescent reporter plasmid containing variable sgRNAs targeting the indicated genes. Cells (>100) were imaged by microscopy after 4 h incubation with (+) and without (-) 0.2% arabinose to induce *dcas9* expression. Normalized P*_cpxP_* reporter activity was compared to that of induced cells expressing no sgRNA (no sgRNA +); cells were considered Cpx^ON^ when normalized reporter activity exceeded twofold the average from the induced no sgRNA control population. One-way ANOVA with multiple comparison test was performed in GraphPad Prism v10.3.0 to determine significance. * p<0.05; ** p<0.01; *** p<0.001; **** p<0.0001. Each sample had 3 biological replicates, except for the *bamA* +, *uppS* +, and *uppS* - conditions, which had 9, 6, and 6 biological replicates, respectively.

**FIGURE S9**


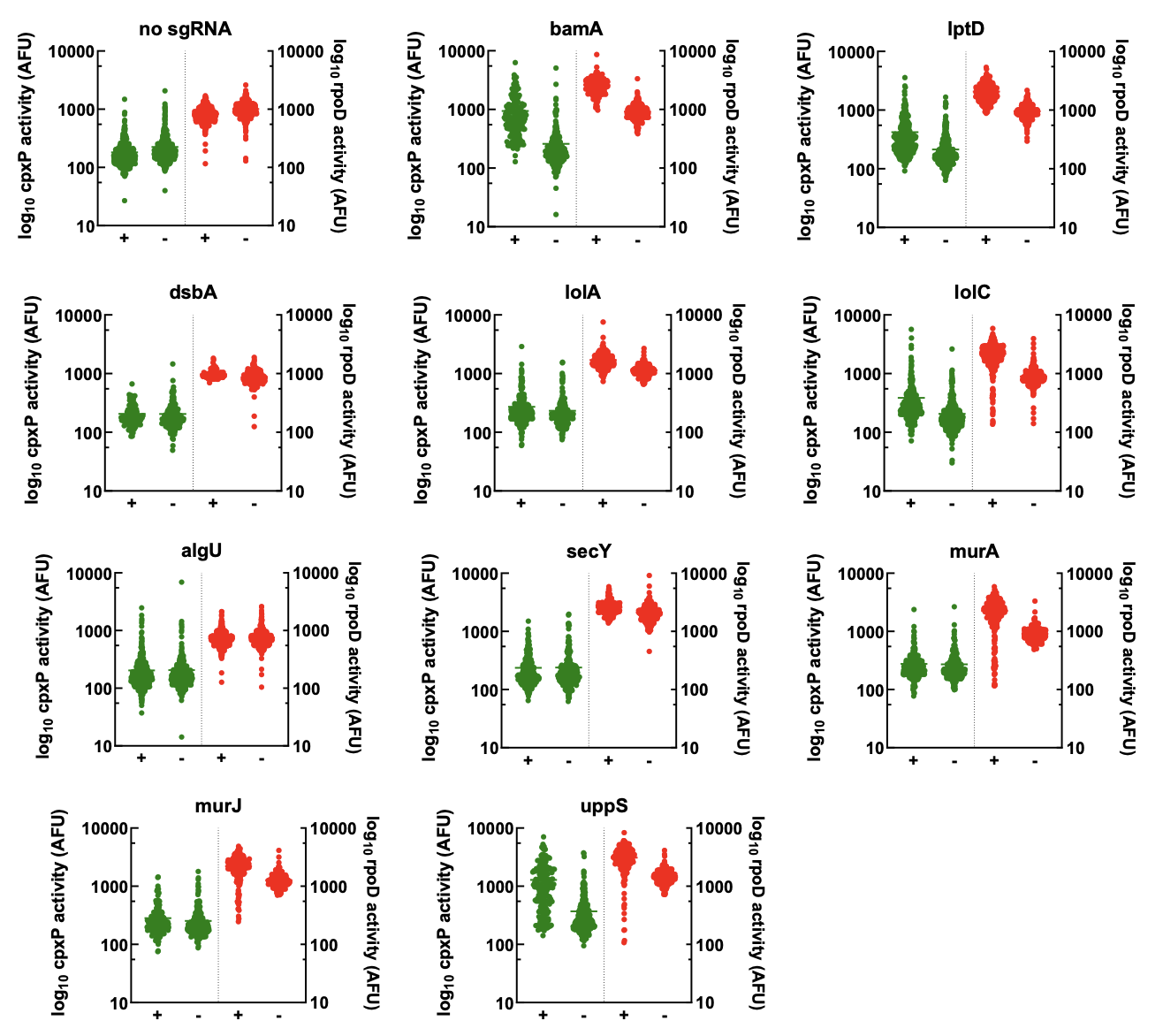


**Fig S9. Raw fluorescence values for individual cells expressing P*_cpxP_*::mGreenLantern/ P*_rpoD_*::mScarlet-I reporters under CRISPRi inducing conditions.** PAO1 strains with an integrated T*n*7::*sp*dcas9 cassette expressed a bicolor P*_cpxP_*::mGreenLantern/P*_rpoD_*::mScarlet-I reporter plasmid. sgRNAs targeting the indicated genes were constitutively expressed from the same plasmid. Cells (>100) were imaged by microscopy after 4 h incubation with (+) and without (-) 0.2% arabinose to induce *sp*dcas9 expression. Graphed are single-cell fluorescence intensity, with background florescence subtracted, with green dots representing P*_cpxP_*::mGreenLantern signal and red dots representing P*_rpoD_*::mScarlet-I signal. Representative samples for each sgRNA are shown.

**FIGURE S10**

***

**Fig S10. Confirmation of a gain-of-function mutation in the PAO1 Cpx signaling system.** Activity of an integrated Tn*7*::P*_cpxP_*::*lacZ* transcriptional reporter was analyzed in PAO1 and a derivative missense point mutant in CpxS (CpxS^T163P^) generated by allelic exchange. Graphed are means (± SD) of P*_cpxP_*::*lacZ* reporter activity quantified by β-galactosidase activity (Miller Units) from cell lysates. Bacterial overnight cultures were diluted and grown 4 h at 37°C in LB broth. Asterisks denote statistical significance based on one-way ANOVA, *** p>0.001. Data are representative of three independent experiments (n=6).

**FIGURE S11**


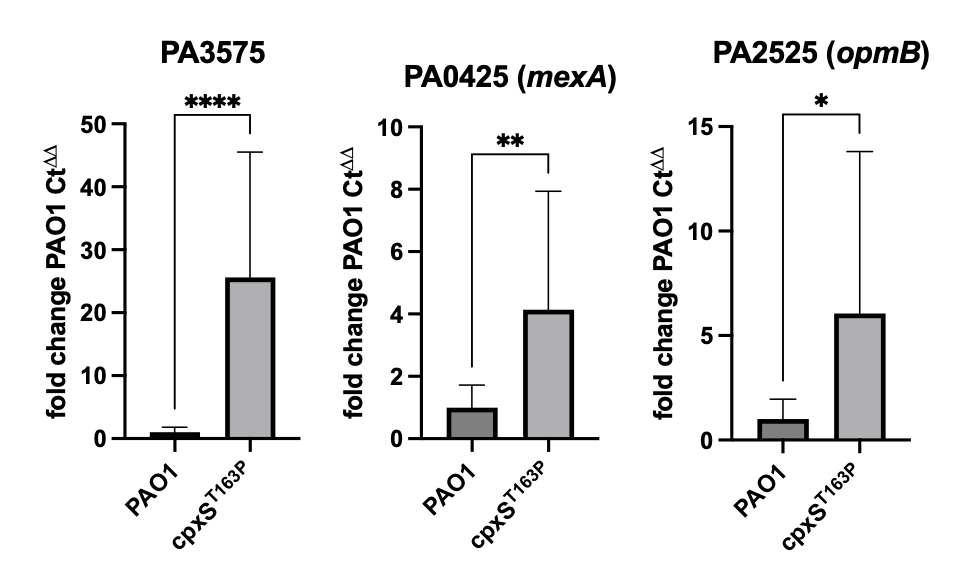


**Fig S11. qRT-PCR validation of differentially expressed genes in PAO1 cpxS^T163P^ identified by RNA-Seq.** PAO1 and PAO1 *cpxS*^T163P^ were diluted from overnight cultures in LB and grown to mid-exponential phase. RNA was purified from frozen cell pellets (~ 1 x 10^9^ cells) and normalized to 3 ng total RNA for cDNA synthesis. qRT-PCR was performed with a BioRad CFX384 thermocycler using the SsoAdvanced Universal SYBR Green Supermix and custom oligonucleotide primers targeting the indicated genes. Real-time amplification was quantified by cycle threshold (Ct) values, called by BioRad CFX Manager. Relative expression of target genes was compared to the housekeeping gene *gyrA* with the standard 2^-∆∆Ct^ formula. Graphed are means (± SD) of fold change values relative to PAO1 (n=8). Four biological replicates and two technical replicates, derived from separate cDNA synthesis reactions from the same purified RNA, were performed per strain. Asterisks indicate statistical significance based on t-test; *** p<0.001; ** p<0.01; * p<0.05.

**FIGURE S12**

**
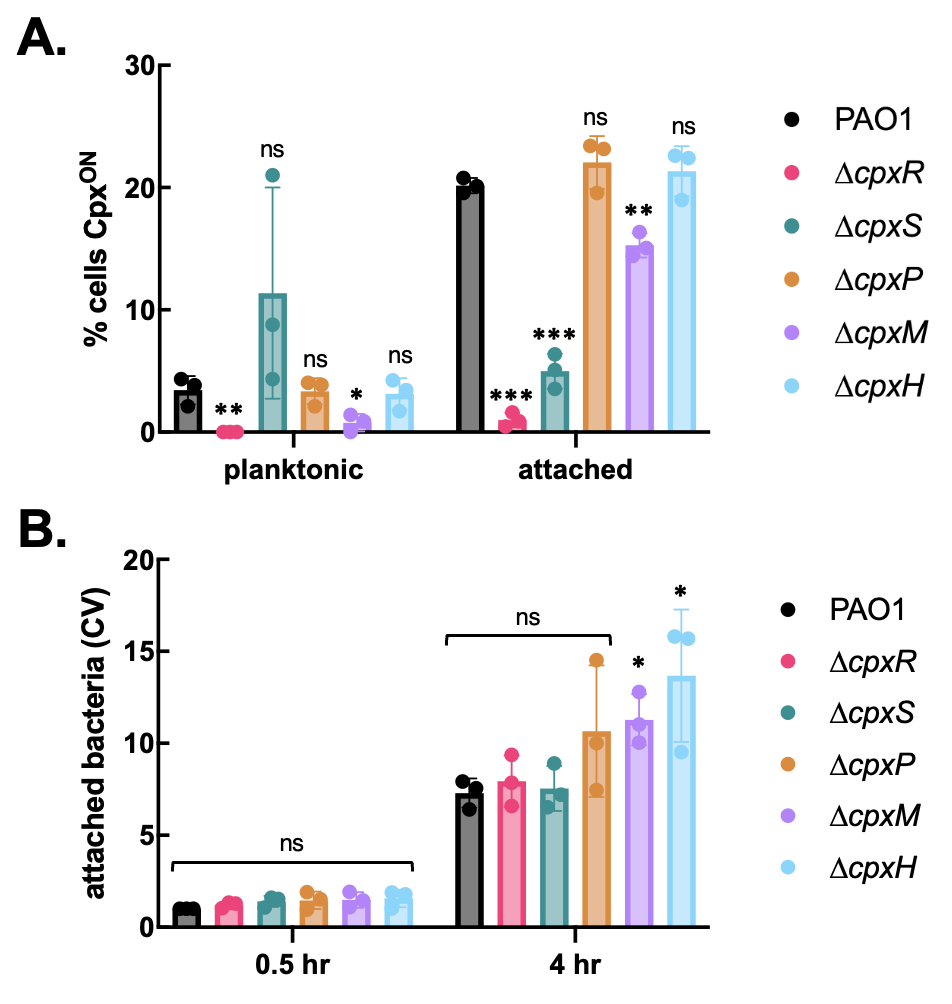
**

**Fig S12. Surface sensing and attachment among PAO1 ∆*cpx* mutants. (A)** Activity of a pBBR1 P_cpxP_::mGreenLantern/P*_rpoD_*::mScarlet-I fluorescent reporter was assessed as previously described among PAO1 and ∆*cpx* mutant strains grown in planktonic culture or exposed to a liquid:agarose surface for 4 h. Individual cells were designated Cpx^ON^ when normalized reporter activity exceeded 5x the average value of the PAO1 ∆*cpxR* planktonic population. Graphed are means (± SD) of the %Cpx^ON^ cells per strain, with asterisks representing statistical significance from pairwise *t*-tests between PAO1 and mutant strains in their respective planktonic and surface-attached growth conditions; “ns” no significant difference, * p < 0.05, ** p < 0.01, *** p < 0.001. (B) Attachment of PAO1 and ∆*cpx* mutant bacterial cultures to a liquid:glass surface. Asterisks denote statistical significance based on pairwise *t*-tests between PAO1 and mutant strain attachment levels at indicated time points; * p < 0.05, “ns” no significant difference . All data are pooled from three independent experiments.

**FIGURE S13**

**
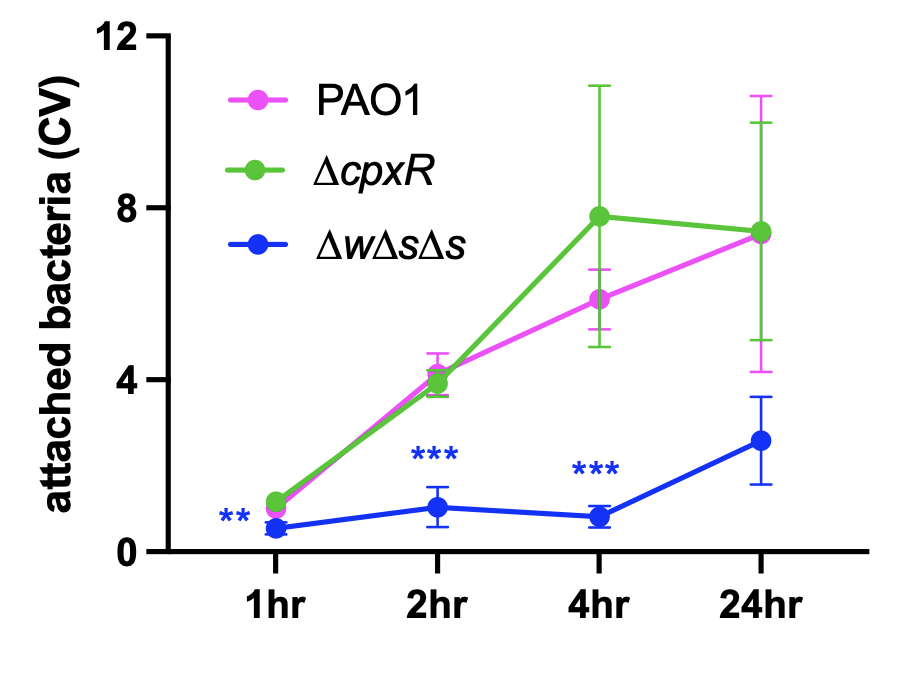
**

**Fig S13. Initial attachment efficiency of PAO1 to glass surfaces is Cpx-independent, but reduced in c-di-GMP-deficient bacteria.** Mid-exponential phase cultures of PAO1 and derivative mutant strains (3h growth in LB) were diluted 1:2 in ddH_2_O and exposed to submerged a glass coverslips. At indicated times, coverslips were removed and stained with 0.5% crystal violet in ddH_2_O to stain attached biomass. Graphed are means (± SD) of attached bacteria measured by destaining coverslips and reading absorbance at 562 nm. Data are normalized to PAO1 attachment levels at 1h. Asterisks denote statistical significance based on pairwise t-tests between PAO1 and mutant strain attachment levels at indicated time points, ** p>0.01, *** p>0.001. Data are pooled from three independent experiments.

**REFERENCES**

1. Karp PD, Billington R, Caspi R, Fulcher CA, Latendresse M, Kothari A, Keseler IM, Krummenacker M, Midford PE, Ong Q, Ong WK, Paley SM, Subhraveti P. 2019. The BioCyc collection of microbial genomes and metabolic pathways. Brief Bioinform 20:1085–1093.

2. Ducret A, Quardokus EM, Brun Y V. 2016. MicrobeJ, a tool for high throughput bacterial cell detection and quantitative analysis. Nat Microbiol 1:1–7.
